# A Comparative Investigation on Phenolic Composition, Characterization and Antioxidant Potentials of Five Different Australian Grown Pear Varieties

**DOI:** 10.3390/antiox10020151

**Published:** 2021-01-20

**Authors:** Zening Wang, Colin J. Barrow, Frank R. Dunshea, Hafiz A. R. Suleria

**Affiliations:** 1 School of Agriculture and Food, Faculty of Veterinary and Agricultural Sciences, The University of Melbourne, Parkville, VIC 3052, Australia; zeningw1@student.unimelb.edu.au (Z.W.); fdunshea@unimelb.edu.au (F.R.D.); 2 Centre for Chemistry and Biotechnology, School of Life and Environmental Sciences, Deakin University, Waurn Ponds, VIC 3217, Australia; colin.barrow@deakin.edu.au; 3 Faculty of Biological Sciences, The University of Leeds, Leeds LS2 9JT, UK

**Keywords:** pear, phenolic compounds, antioxidant activity, LC-MS/MS, HPLC-PDA

## Abstract

Pear (*Pyrus communis* L.) is widely spread throughout the temperate regions of the world, such as China, America and Australia. This fruit is popular among consumers due to its excellent taste and perceived health benefits. Various bioactive compounds, which contribute to these health benefits, have been detected in the pear fruits, including a range of phenolic compounds. Five Australian grown pear varieties, which include Packham’s Triumph, Josephine de Malines, Beurre Bosc, Winter Nelis and Rico were selected for this study to examine the phenolic compounds in pears. Beurre Bosc exhibited the highest total polyphenol content (TPC) (3.14 ± 0.02 mg GAE/g), total tannin content (TTC) (1.43 ± 0.04 mg CE/g) and 2,2′-diphenyl-1-picrylhydrazyl (DPPH) (5.72 ± 0.11 mg AAE/g), while the Josephine de Malines variety was high in total flavonoid content (TFC) (1.53 ± 0.09 mg QE/g), ferric reducing antioxidant power (FRAP) (4.37 ± 0.04 mg AAE/g), 2,2′-azinobis-(3-ethylbenzothiazoline-6-sulfonic acid) (ABTS) (4.44 ± 0.01 mg AAE/g) and total antioxidant capacity (TAC) (5.29 ± 0.09 mg AAE/g). The liquid chromatography coupled with electrospray-ionization quadrupole time-of-flight mass spectrometry (LC-ESI-QTOF-MS/MS) data indicate that a total of 73 phenolic compounds were detected in Beurre Bosc (37 compounds), Josephine de Malines (34), Rico (22), Packham’s Triumph (15) and Winter Nelis (9), respectively. From HPLC-PDA quantification, the Beurre Bosc pear variety showed significantly higher in phenolic acids (chlorogenic acid; 17.58 ± 0.88 mg/g) and while flavonoids were significantly higher in Josephine de Malines (catechin; 17.45 ± 1.39 mg/g), as compared to other pear varieties. The analyses suggest that the Australian grown pears might contain an ideal source of phenolic compounds which benefit human health. The information provided by the present work can serve as practical supporting data for the use of pears in the nutraceutical, pharmaceutical and food industries.

## 1. Introduction

Pears (*Pyrus communis* L.) are one of the most common fruits in people’s daily life. Pears are not only delicious and cheap, but they are also rich in phytochemicals [1]. Pears are grown in the temperate regions of the world which includes more than 50 countries. In 2019, the global production of pears reached 23.1 million tons [2] with around 10% of pears production being processed into various products, such as canned pears, concentrated pear juice and fresh cut pears [3]. Pears are rich sources of bioactive compounds such as phytochemicals, soluble sugars, amino acids, vitamins and minerals [4]. Bioactive compounds extracted from different pears are reported as being protective against various human disorders including ageing, cancer, cardiovascular disease, nerve dysfunction, respiratory distress syndrome and diabetes [5]. Thus, the bioactive compound extracts from different pears, especially polyphenols, may contribute to human health.

Polyphenols are important plant-derived secondary metabolites, which include hydrolyzed tannin (acid ester polyphenols) and condensed tannin (flavanols polyphenols or proanthocyanins) [6]. Polyphenols consist of aromatic rings with attached hydroxyl groups, organic acids and acylated sugars. This unique structure endows polyphenols with high antioxidant activity which can directly or indirectly prevent the formation of free radicals [5]. The most abundant polyphenols in pears include flavan-3-ols, flavonols, phenolic acids, anthocyanins and hydroquinones [7]. The antioxidant potential of polyphenols in pears can be determined by several chemical assays each of which depend upon different mechanisms. These in vitro spectrophotometric-based assays include 2,2′-diphenyl-1-picrylhydrazyl (DPPH) free radical scavenging assay, ferric reducing antioxidant power (FRAP) assay, 2,2′-azinobis-(3-ethylbenzothiazoline-6-sulfonic acid) (ABTS) assay and total antioxidant capacity (TAC) [8].

In recent years, there has been increasing interest in the extraction of polyphenols from different plant materials. It is difficult to extract, separate, determine and identify a particular type of phenolic compounds from plant-materials due to their chemical and structural diversity. Liquid chromatography coupled with electrospray-ionization quadrupole time-of-flight mass spectrometry (LC-ESI-QTOF-MS/MS), is a new technique with higher sensitivity, which is purported to be the most effective method for the characterization and determination of both low and high molecule weight polyphenols [9]. Furthermore, high performance liquid chromatography (HPLC) is another useful tool that is used to quantify the targeted phenolic compounds in combination with different detectors, such as ultraviolet–visible (UV) and photodiode array detector (PDA) [10]. In the previous study, the phenolic compounds, which include quinic acid, flavan-3-ols, flavonols, flavones, hydroquinones, anthocyanins and their derivatives were characterized using both HPLC and LC-MS methods [1]. These results show that caffeic acid, monomeric catechins, polymeric procyanidins, isorhamnetin derivatives, chlorogenic acid and arbutin are the main phenolic compounds in pears [1].

Although a number of studies have identified and quantified the phenolic compounds in different pear varieties grown in different regions, only a few of them focus on the phenolic compounds in Australian grown pears, which include Packham’s Triumph, Josephine de Malines, Beurre Bosc, Winter Nelis and Rico. Therefore, the objective of this study is to determine the total polyphenol content (TPC), total flavonoid content (TFC) and total tannin content (TTC) in Australian grown pears and measure the antioxidant activity by determining DPPH, ABTS radical-scavenging activity, FRAP and TAC. This study also characterized and identified the phenolic compounds from pears by LC-ESI-QTOF-MS/MS and quantified through HPLC-PDA.

## 2. Materials and Methods

### 2.1. Chemicals and Reagents

Most of the chemicals used for extraction and characterization were analytical grade and purchased from Sigma-Aldrich (Castle Hill, NSW, Australia). Folin and Ciocalteu’s phenol reagent, gallic acid, L-ascorbic acid, vanillin, hexahydrate aluminum chloride, quercetin, catechin, 2,2′-diphenyl-1-picrylhy-drazyl (DPPH), 2,4,6-tripyridyl-s-triazine (TPTZ) and 3-ethylbenzothiazoline-6-sulphonic acid (ABTS) were bought from the Sigma-Aldrich (Castle Hill, NSW, Australia). The chemical reagent and reference standard for HPLC, including gallic acid, protocatechuic acid, *p*-hydroxybenzoic acid, chlorogenic acid, caffeic acid, catechin, epicatechin, epicatechin gallate, quercetin and kaempferol were produced by Sigma-Aldrich (Castle Hill, NSW, Australia). Sodium carbonate anhydrous were purchased from Chem-Supply Pty Ltd. (Adelaide, SA, Australia) and 98% sulfuric acid were bought from RCI Labscan (Rongmuang, Thailand). Methanol, acetonitrile, ferric chloride (Fe[III]Cl_3_•6H_2_O), hydrated sodium acetate, hydrochloric acid and glacial acetic acid were purchased from Thermo Fisher Scientific Inc. (Scoresby, VIC, Australia).

### 2.2. Sample Preparation

**Figure 1:**
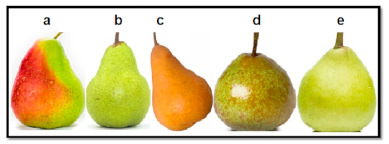
Australian grown pear varieties (**a**), Rico (**b**), Packham’s Triumph (**c**), Beurre Bosc (**d**) Winter Nelis and (**e**) Josephine de Malines.

The fresh fruits of five Australian grown pear varieties, Rico, Packham’s Triumph, Beurre Bosc, Winter Nelis and Josephine de Malines (Figure 1) grown in different region of Victoria were purchased from local markets in Melbourne, Victoria, Australia. A fully mature pear samples were harvested, stored at room temperature for 24–48 h for optimum ripening followed by transportation and distribution to the local retailers within 2–3 days using refrigerated trucks. Two to three kilogram samples of each variety were cleaned and peeled; the seeds were removed and pulp was blended using the 1.5 L blender (Russell Hobbs Classic, model DZ-1613, Melbourne, VIC, Australia). The pear pulps were kept at −20 °C for 48 h and lyophilized at −45 °C/50 MPa by Dynavac engineering FD3 Freeze Drier (W.A., Australia) and Edwards RV12 oil sealed rotary vane pump (Bolton, UK) and the freeze dried powders were stored at −20 °C.

### 2.3. Extraction of Phenolic Compounds

The extracts were made by modifying the protocol of Peng et al. [10], 5 g of pear powder was mixed with 20 mL 70% ethanol and homogenized with the IKA Ultra-Turrax^®^ T25 homogenizer (Rawang, Selangor, Malaysia) and subjected to shaking incubator (ZWYR-240, Labwit, Ashwood, VIC, Australia) at 120 rpm for 12 h (4 °C). After incubation, the pear extracts were centrifuged with Hettich Refrigerated Centrifuge (ROTINA380R, Tuttlingen, Baden-Württemberg, Germany) at 1000 rpm for 15 min. The supernatants were collected and stored at −20 °C before further analysis.

### 2.4. Estimation of Phenolic Compounds and Antioxidant Assay

The TPC, TFC and TTC assays were conducted to estimate the polyphenols in the samples. For antioxidant activities, three different antioxidant assays, including DPPH, ABTS and FRAP assay were used, which are based on the method and parameter from Tang et al. [11]. The spectrophotometric data were collected on the Multiskan^®^ Go microplate photometer (Thermo Fisher Scientific, Waltham, MA, USA).

#### 2.4.1. Determination of Total Phenolic Content (TPC)

The TPC of pear samples was determined by the spectrophotometric method of Samsonowicz et al. [12] with some modification. The sample extracts (25 µL), Folin–Ciocalteu reagent solution (25 µL, 1:3 diluted with water) and Milli-Q water (200 µL) were added in a 96-well plate (Costar, Corning, NY, USA). After incubation (25 °C, 5 min), 25 µL 10% (*w*:*w*) sodium carbonate was added and followed by incubation in the dark for 60 min. The absorbance was determined at 764 nm in a microplate reader (Thermo Fisher Scientific, Waltham, MA, USA). The quantification of each sample was based on the standard curve that was generated with 0–200 µg/mL gallic acid in ethanol. The result was expressed as mg of gallic acid equivalents per gram dry weight of sample (mg GAE/g of DW).

#### 2.4.2. Determination of Total Flavonoid Content (TFC)

The TFC of the pear sample was measured by modifying the aluminum chloride method of Stavrou et al. [13]. The sample extracts (80 µL), 2% aluminum chloride (80 µL, *w*/*v*, diluted with ethanol) and sodium acetate solution (120 µL, 50 g/L) were mixed in a 96-well plate and then incubated in the dark at room temperature for 60 min. The absorbance was measured at 440 nm in a microplate reader. The calculation of TFC of each sample was based on the standard curve of quercetin (0 to 50 µg/mL) and the result was expressed as mg of quercetin equivalent per g (mg QE/g DW) of dry weight.

#### 2.4.3. Determination of Total Tannin Content (TTC)

The TTC of the pear samples was estimated by the method of Stavrou et al. [13] with some modification. The sample extracts (25 µL, 1:50 diluted with methanol), 4% vanillin solution (150 µL, diluted with methanol) and 32% sulfuric acid (25 µL) were mixed in a 96-well plate and followed by incubation at 25 °C for 15 min. The absorbance was measured at 500 nm against a blank in a microplate reader. The calculation of results was based on the standard curve of catechin solution (concentration from 0–1000 µg/mL). The result was expressed as mg of catechin equivalent per g (mg CE/g DW) of dry weight.

#### 2.4.4. 2,2′-Diphenyl-1-picrylhydrazyl (DPPH) Assay

The radical scavenging activity of pears was determined by DPPH assay method with some modification of Sogi et al. [14]. Sample extract (40 µL) was mixed with 0.1 M DPPH radical methanol solution (260 µL) in a 96-well plate, then incubated at 25 °C for 30 min. The absorbance was measured at 517 nm in a microplate reader. The standard curve was generated with different concentration of ascorbic acid (0 to 50 µg/mL). The result was expressed as mg ascorbic acid equivalents per g of dry weights (mg AAE/g DW).

#### 2.4.5. Ferric Reducing Antioxidant Power (FRAP) Assay

The reducing capacity of the pear samples was determined based on the method of Sogi et al. [14] with some modification. This method involved the determination of the ability to reduce Fe^3+^ in the Fe^3+^-TPTZ complex (ferric-2,4,6-tripyridyl-s-Triazine) into Fe^2+^-TPTZ. Sodium acetate solution (300 mM), TPTZ solution (10 mM) and ferric chloride (20 mM) was mixed in a ratio of 10:1:1 (*v*/*v*/*v*) to freshly made FRAP reagent. Then, sample extract (20 µL) and prepared FRAP dye solution (280 µL) were added to a 96-well plate and followed by incubation at 37 °C for 10 min. The absorbance was measured at 593 nm in microplate reader. The standard curve was generated with ascorbic acids range from 0 to 50 µg/mL. The results were expressed as mg of ascorbic acid equivalents per g (mg AAE/g DW) of dry weight.

#### 2.4.6. 2,2′-Azino-bis-3-ethylbenzothiazoline-6-sulfonic Acid (ABTS) Assay

The free radical scavenging capacity of the pear samples was determined by ABTS^+^ radical cation decolorization assay with some modification of Sogi et al. [14]. 7 mmol/L of ABTS solution (5 mL) and 140 mM potassium persulfate decolorization solution were mixed to make ABTS^+^ stock solutions. The mixture was incubated in the dark for 16 h (room temperature) and then ABTS^+^ stock solution was diluted with ethanol to obtain an initial absorbance of 0.700 at 734 nm. Afterwards, the pear sample extract (10 µL) was mixed with the prepared diluent in 96-well plate and followed by incubation in the dark at room temperature for 6 min. The absorbance was measured at 734 nm in microplate reader. The standard curve was generated with ascorbic acid with concentration ranging from 0 to 150 µg/mL. The ABTS value was expressed as mg of ascorbic acid equivalents per g (mg AAE/g DW) of dry weight.

#### 2.4.7. Total Antioxidant Capacity (TAC)

The TAC was determined by following the modified protocol of Subbiah et al. [15]. First, 0.6 M H_2_SO_4_, 0.028 M sodium phosphate and 0.004 M ammonium molybdate were mixed to make the phosphomolybdate reagent. Next, the pear sample extract (40 µL) and phosphomolybdate reagent (260 µL) were mixed in 96-well plate and followed by incubation at 95 °C for 10 min. The absorbance was determined at 695 nm. The standard curve was generated with ascorbic acid with concentration ranging from 0–200 μg/mL. The TAC values were expressed as mg of ascorbic acid equivalents per g (mg AAE/g DW) of dry weight.

### 2.5. Characterization of Phenolic Compounds by LC-ESI-QTOF-MS/MS Analysis

Characterization of phenolic compounds in pears was carried out using the method of Suleria et al. [16]. An Agilent 1200 series HPLC (Agilent Technologies, Santa Clara, CA, USA) equipped with Agilent 6520 I Accurate-Mass Q-TOF LC-MS/MS (Agilent Technologies, Santa Clara, CA, USA) was used in the characterization and identification of phenolic compounds in pears. The separation of different phenolic compounds was conducted in Synergi Hydro-RP 80Å LC reverse phase with 250 mm × 4.6 mm internal diameter and 4 µm particle size (Phenomenex, Torrance, CA, USA). The mobile phase A, which consists of water/acetic acid solution (98:2, *v*/*v*), was combined with mobile phase B, which consists of acetonitrile/water/acetic acid solution (50:49.5:0.5, *v*/*v*/*v*) to form the binary solvent system at the flow rate of 0.8 mL/min with a sample injection volume 6 µL. The gradient elution lasted for 85 min with the following conditions: 0 min, 90% A and 10% B; 20 min, 75% A and 25% B; 30 min, 65% A and 35% B; 40 min, 60% A and 40% B; 70 min, 45% A and 55% B; 75 min, 20% A and 80% B; 77–79 min, 0% A and 100% B; 82–85 min, 90% A and 10% B. Both the positive and negative ion mode were used in peak identification with capillary at 3.5 LV and nozzle voltage at 500 V. The parameters of mass spectrometry were set as follows: for nitrogen gas, the pressure was set at 45 psi with the flow rate of 5 L/min at 300 °C; for sheath gas, the flow rate and temperature were set at 11 L/min and 250 °C. The range of mass spectra was set at *m/z* 50–1300. Further, MS/MS analyses were carried out in automatic mode with collision energy (10, 15 and 30 eV) for fragmentation. Data collection and processing was conducted using MassHunter (Qualitative Analysis, version B.03.01, Agilent).

### 2.6. HPLC–PDA Analysis

The quantification of targeted phenolic compounds in pears was measured by the method of Zhong et al. [17] and performed by Agilent 1200 series HPLC (Agilent Technologies, Santa Clara, CA, USA) equipped with a photodiode array (PDA) detector. The column and conditions in HPLC-PDA analysis were similar to LC-MS/MS analysis, while the sample injection volume was changed to 20 µL. The phenolic compounds were determined at three different wavelengths, including 280 nm, 320 nm and 370 nm. The quantification of the concentration of individual polyphenols was based on the calibration standard curve and the result was expressed as mg/g of sample. Data collection and processing was performed using Agilent LC-ESI-QTOF-MS/MS MassHunter (Qualitative Analysis, version B.03.01, Agilent).

### 2.7. Statistical Analysis

Three parallel experiments were conducted in each test and the data are expressed as mean ± standard deviation. Statistical analysis was performed by using Minitab^®^ 18 Statistical software (Minitab Inc., State College, PA, USA). One-way analysis of variance (ANOVA) followed by Tukey’s honestly significant differences (HSD) multiple rank test was carried out to test whether there was a significant difference between the antioxidant activities and polyphenol content of each sample at *p* < 0.05.

## 3. Results and Discussion

### 3.1. Phenolic Compound Estimation (TPC, TFC and TTC)

Previously, several studies have estimated the phenolic content of different pear varieties by determining different parameters. In this study, the phenolic content in pears were determined by the TPC, TFC and TTC and the results were expressed as mg of gallic acid equivalents per gram dry weight of sample (mg GAE/g of DW) mentioned in Table 1.

The TPC of Beurre Bosc pulp (3.14 ± 0.02 mg GAE/g) was higher than Josephine de Malines, Packham’s Triumph, Winter Nelis and Rico. Our results were slightly higher than Turkish grown pear varieties including Egirsah, Gugum, Deveci, Kizil and Banda, also extracted in methanol with different concentration (1.75 ± 0.13 mg GAE/g) [18]. The difference in the sample variety, growing region, sample extraction techniques, type of solvent, solute to solvent ratio may contribute to the difference in results. However, Manzoor et al. [19] reported the total phenolic content of Nakh and Naspati pear were in range of our study.

Regarding the TFC, the Josephine de Malines (1.53 ± 0.09 mg QE/g DW) had higher flavonoid content than Beurre Bosc, Packham’s Triumph, Winter Nelis and Rico. The results were similar to the TFC values (ranging from 0.3 to 6 mg QE/g) reported by Azzini et al. [20] and Li et al. [21]. Rawat et al. [22] also determined the higher total flavonoid content in one of the southern Asia grown pear varieties (*Pyrus pashia*) by aluminum chloride colorimetric assay and extracted with different solvent and solvent to solute ratio. Previously, Patricia et al. [23] also confirmed that the total flavonoid content of pears can be varied significantly using different extraction solvents including *n*-hexane, ethyl acetate, ethanol and methanol.

The Beurre Bosc pear has the highest TTC value (1.43 ± 0.04 mg CE/g DW) as compared to other pear varieties (Table 1). Previously, only a few studies have focused on the total tannin content of pears. Ma et al. [24] have detected slightly higher total tannin content in one of the apple-shaped pear varieties (*Pyrus pyrifolia*) using another method of *n*-BuOH-HCl-Fe-III. In addition, Velmurugan and Bhargava [25] had also reported difference in total tannin content of European grown pear varieties extracted with different solvents (chloroform, aqueous, ethyl acetate and ethanol) and using another method of Folin–Denis. However, due to the difference in the detection methods and calibrators, it is difficult to be compare these findings with the TTC value in our experiment. This is why we have expanded our suite of antioxidant assays to better characterize the antioxidant potential of Australian grown pear samples.

### 3.2. Antioxidant Activity (DPPH, FRAP, ABTS and TAC)

The antioxidant activities of pears were determined by DPPH, FRAP, ABTS and TAC assays. In current research, different types of antioxidant assays were performed, involved different mechanisms, to understand overall and true antioxidant potential of pear fruit. The results of DPPH test ranged from 3.25 ± 0.03 to 5.72 ± 0.1 mg AAE/g, with statistically significant differences between pear varieties except for Josephine de Malines and Rico (Table 1). The Beurre Bosc had the highest DPPH free radical scavenging capacity followed by Josephine de Malines, Rico, Packham’s Triumph and Winter Nelis. In the previous study, Nomura et al. [26] measured the DPPH radical scavenging activity of 29 varieties of Japanese and European grown pears, some of their pear varieties had the similar DPPH free radical scavenging activity with our Australian grown pear varieties. In addition, Galvis Sánchez et al. [27] determined the DPPH free radical scavenging capacity of six Chile grown European pear varieties while their DPPH values were slightly lower than our study. The difference in pear varieties, extraction solvent, growing region, condition and harvesting season may contribute to the difference of results.

For the FRAP assay, all the varieties show significant differences from each other. Josephine de Malines had the highest FRAP activity which is 4.37 ± 0.04 mg AAE/g, followed by Beurre Bosc, Packham’s Triumph, Rico and Winter Nelis. Previously, the total antioxidant activity of two Greece grown pears varieties (Naoussa and Vergina) measured by FRAP method ranged from 1.41 mg AAE/g to 1.93 mg AAE/g which is slightly lower than our FRAP values [28,29]. Gu et al. [30] also reported the slightly lower antioxidant capacity of European pears extracted with different solvent to solute ratio. However, Jamuna et al. [31] detected the antioxidant activity of an Indian grown pear variety (*Pyrus communis*) by FRAP assay and the result (3.00 mg AAE/g) was consistent with our Australian grown pear varieties.

Based on the ABTS assay, Josephine de Malines and Beurre Bosc were not different, but they were significantly higher than the other varieties. Thus, Josephine de Malines and Beurre Bosc have the highest ABTS value (4.44 ± 0.01 and 4.41 ± 0.07 mg AAE/g, respectively) followed by Packham’s Triumph, Rico and Winter Nelis (Table 1). In the previous study, Batista et al. [32] reported that the ABTS values of different Portugal grown pear varieties including S. Bartolomeu and Amêndoa pears and the results were slightly lower than our investigation. Erbil et al. [18] also measured the ABTS free radical scavenging activity of five different Turkish grown pear varieties (Egirsah, Gugum, Deveci, Kizil and Banda) and found that there was a significant difference of the ABTS free radical scavenging capacity between different pear varieties grown in the same region.

When antioxidant activity was measured using the TAC assay there were significant differences between all varieties except Beurre Bosc and Winter Nelis. Josephine de Malines had the higher TAC value (5.29 ± 0.09 mg AAE/g), followed by Packham’s Triumph, Winter Nelis, Beurre Bosc and Rico. Previously, Jamuna et al. [33] reported the TAC capacity (1.45 mg AAE/g) of Indian grown pear variety (*Pyrus communis*) extracted using chloroform-water which was lower than our values. The difference in pear varieties, growing region, extraction solvent, solute to solvent ratio and harvesting season may contribute to the difference of results.

In the present study, Beurre Bosc had the highest antioxidant capacity when measured using TPC, TTC, DPPH, FRAP and ABTS assays while Winter Nelis was consistently low TAC for all assays. Thus, the antioxidant capacity of pears can be associated with their polyphenol content. Different varieties, genotypes and agronomy of pears influence the polyphenol content and thereby influence their antioxidant activity.

### 3.3. Correlation among Different Antioxidant Variables

The correlations between TPC, TFC, TTC and antioxidant assays (DPPH, FRAP, ABTS and TAC) were performed by Pearson’s correlation test by Graphpad Prism 8 (Table 2). There was a significant positive correlation between TPC and TTC (*r* = 0.93, *p* ≤ 0.05) and a highly significant positive correlation between TPC and DPPH value (*r* = 0.97, *p* ≤ 0.01). This result is consistent with the research reported by Kolniak-Ostek [1]. The TPC estimate the phenolic content in samples and DPPH scavenging capacity measures the antioxidant activities, therefore the phenolic content in pears mainly contribute to the antioxidant activities of sample.

Furthermore, TFC is strongly correlated with FRAP value with Pearson’s correlation coefficient (*r* = 0.93, *p* ≤ 0.01). Previously, the strong positive correlation between TFC and FRAP value of pear was also observed by Azzini et al. [20] with *r* = 0.919. FRAP measures the ability to reduce the Fe^3+^-TPTZ complex into Fe^2+^-TPTZ, while TFC only determined the flavonoid content in samples. Thus, this correlation indicates that the antioxidant capacity is positively related to the presence of flavonoid compounds. Furthermore there was also a significant positive correlation between TFC and ABTS (*r* = 0.87, *p* ≤ 0.05) which was similar to the findings of Floegel et al. [34]. However, Li et al. [21] also observed a strong correlation between DPPH and TFC which was not significant in this study. This may due to the flavonoid compounds have no obvious effect on DPPH free radical scavenging ability.

Apart from above, TTC displays a highly significant positive correlation with DPPH assay with (*r* = 0.96, *p* ≤ 0.01) which was similar to the findings of Li et al. [21]. This strong correlation indicates that the tannin in pears were related to the DPPH free radical scavenging capacity. This may due to the molecular structure of tannin in pears can provide H to DPPH free radicals to form DPPH-H [21]. Additionally, the FRAP assay is highly correlated with ABTS assay in the present study (*r* = 0.94, *p* ≤ 0.01) which was consistent with the findings of Du et al. [35] who also observed a strong positive correlation (*r* = 0.92) between TAC measured using the FRAP and ABTS methods. The strong correlations between TAC measured using ABTS and FRAP is expected because both methods are relying on the single electron transfer principle. For further elucidation of the sources of the TAC activity, LC-ESI-QTOF-MS/MS and HPLC-PDA were used for identification, characterization and quantification of phenolic compounds in different pear samples.

### 3.4. LC-MS Analysis

In the present work, the quantitative analysis of the phenolic compounds in five varieties of pears was carried out by LC-ESI-QTOF-MS/MS analysis in both positive and negative ionization modes. The phenolic compounds were tentatively identified and characterized and based on the *m/z* value from MS spectra in both negative and positive modes ([M − H] ^−^/[M + H]^+^) (Supplementary data: Figures S1 and S2). The data analysis was carried out by using Agilent LC/MS Mass Hunter Qualitative software and Personal Compound Database and Library (PCDL). Only the compounds with a PCDL score higher than 80 and the mass error less than ±5 ppm were selected for characterization and verification purposes. A total of 73 phenolic compounds were identified in different pear varieties, which include phenolic acids (30), flavonoids (29), stilbenes (1) and other polyphenols (13) mentioned in Table 3.

#### 3.4.1. Phenolic Acids

In this study, 4 classes of phenolic acids were detected in five pear samples (Table 3) resulting in two dominant subclasses of phenolic acids in pear samples being hydroxybenzoic acids (eight phenolic acids) and hydroxycinnamic acids (17), respectively. In addition, two hydroxyphenylacetic acids and three hydroxyphenylpropanoic acids were also identified in pear samples.

##### Hydroxybenzoic Acids

Hydroxybenzoic acids are widely present in different fruits and they are not only capable of inhibit α-amylase and α-glucosidase activity, but also reduce the enzymes responsible for breaking down complex carbohydrates, which help to control glycemia in humans [36]. According to the LC-MS results, three out of the eight different hydroxybenzoic acids were identified in only one pear sample (Compounds **1**, **3**, **7**). Compounds **3** and **5** were both detected all pear varieties except Winter Nelis. Compound **3** was tentatively identified as gallic acid with the precursor ion [M − H] ^−^ at *m/z* 169.0145 and further confirmed by the MS^2^ experiment which shows a loss of CO_2_ (44 Da) at *m/z* 125 [37]. Furthermore, two gallic acid derivatives, which are gallic acid 3-*O*-gallate (compound **7**) and gallic acid 4-*O*-gallate (compound **2**) were characterized based on the precursor ion at *m/z* 321.0239 and *m/z* 331.0675, respectively. According to the result of the MS^2^ experiment, gallic acid 3-*O*-gallate shows a loss of galloyl moiety (152 Da) from the precursor ion at *m/z* 169, while gallic acid 4-*O*-gallate displays a loss of the glucoside moiety (162 Da) and consecutive loss of CO_2_ (44 Da) from the precursor ion at *m/z* 169 and 125 [38,39].

In a previous study, the appearance of gallic acid in the peel and pulp of different pear varieties have been reported by Yim and Nam [4] and Li et al. [21]. Additionally, some hydroxybenzoic acids were characterized by several studies on different fruits or vegetables. For example, Rajauria et al. [38] identified the gallic acid 4-*O*-glucoside in *Himanthalia elongata* Irish seaweed by LC-DAD-ESI-MS/MS and using 60% methanol as the extracting agent. Catarino et al. [40] identified the protocatechuic-acid 4-*O*-glucoside in the stems of *Eriocephalus africanus* by LC-DAD-ESI/MS^n^ method. 2-Hydroxybenzoic acid, which is also known as salicylic acid, was identified not only in pears, but also in other fruits, including kiwifruit and grapes [41]. In the previous study, vanillic acid 4-sulfate and 2, 3-dihydroxybenzoic acid were identified by Zhong et al. [17] and Pj et al. [42] in seaweed and *Flacourtia inermis* fruit, respectively.

##### Hydroxycinnamic Acids

Only 6 out of 17 different hydroxycinnamic acids (Compounds **9**, **10**, **12**, **15**, **19**, **20**) were detected in more than one pear sample. Among them, compounds **10**, **12**, **19**, **20** were identified in both positive and negative modes. Compound **22** was tentatively identified as ferulic acid according to the precursor ion [M − H] ^−^ at *m/z* 193.0499. In the MS^2^ experiment of compound **22**, the product ion at *m/z* 178, *m/z* 149 and *m/z* 134 were due to the loss of CH_3_, CO_2_ and CH_3_ with CO_2_ from the precursor, respectively [43]. Caffeic acid (Compound **12**) was observed in with [M − H]^−^ *m/z* at 179.0346 in both the negative and positive ionization mode. The identification of caffeic acid was achieved by the MS^2^ experiment which displayed the product ions at *m/z* 143 and *m/z* 133 result from the loss of 2 water (36 Da) and HCOOH (46 Da), respectively [44]. Compound **10**, which was present in all pear varieties except Josephine de Malines, was tentatively detected as cinnamic acid based on the precursor ion [M − H]^−^ *m/z* at 147.0461 in both the negative and positive ionization modes and further confirmed by the MS^2^ experiment which shows a loss of CO_2_ (44 Da) at *m/z* 103 [45].

Previously, the appearance of cinnamic acid in 10 different pear varieties had been reported by Sun et al. [46]. Furthermore, the presence of caffeic and *m*-coumaric acid in the flesh and peel of pear varieties were also determined by Öztürk et al. [47]. Simirgiotis et al. [48] identified caffeoyl glucose in the peel of the small Easter Pear. In an earlier study, ferulic acid was been identified in five different pear varieties [49,50]. Regarding other plants, Suleria et al. [16] characterized the feruloyl tartaric acid in pear peels and other fruit peels. In addition, Lin and Harnly [51] confirmed the appearance of the 3-caffeoylquinic acids in the pear skin. According to Ludwig et al. [52], the ferulic acid 4-*O*-glucuronide has been detected in the red raspberry by UHPLC-MS analysis. Piovesana and Noreña [53] isolated 3-*p*-coumaroylquinic acid from *Hibiscus sabdariffa* L. by HPLC-QTOF-MS method. In addition, coffee and stone fruit was also reported as a good source of 3-feruloylquinic acid by the previous study [54]. Regarding the rosmarinic acid, several studies have reported the presence of this compound. Hossain et al. [55] identified rosmarinic acid in Lamiaceae herbs with precursor ion [M − H] ^−^ at *m/z* 359.0763 and the result of MS/MS is at *m/z* 197, 179, 161 and 135 by the LC-ESI-MS/MS method. Chaowuttikul et al. [56] also characterized the rosmarinic acid in some Thai medicinal plants by the RP-HPLC-DAD method and found that the concentration is highest in *Melissa officinalis* leaves. In addition, some compounds, such as caffeic acid 3′-*O*-glucuronide and *p*-coumaroyl tartaric acid were first reported here in pears to our best knowledge.

##### Hydroxyphenylacetic Acids and Hydroxyphenylpropanoic Acids

Only 2 hydroxyphenylacetic acids and 3 hydroxyphenylpropanoic acids were characterized in pear samples. Compound **29** and compound **30** were both detected in the negative ionization mode with the precursor ion [M − H] ^−^ at *m/z* 357.0828 and *m/z* 371.0975 and characterized as dihydrocaffeic acid 3-*O*-glucuronide and dihydroferulic acid 4-*O*-glucuronide. According to the MS^2^ spectra, dihydrocaffeic acid 3-*O*-glucuronide and dihydroferulic acid 4-*O*-glucuronide showed the fragments at *m/z* 181 and *m/z* 195, respectively and the characteristic loss of glucuronide (176 Da) moiety was observed in both compounds [57]. In a previous study, Cuadrado-Silva et al. [58] quantified only trace amounts of 3,4-dihydroxyphenylacetic acid in *P. friedrichsthalianum* fruit by the UHPLC-ESI/QqQ method. Trautvetter et al. [59] also identified this compound in five varieties of honey at *m/z* 167.0344 by the UPLC-MS method. However, to our best knowledge the present study is the first to report the presence of 3-hydroxy-3-(3-hydroxyphenyl) propionic acid in pears.

#### 3.4.2. Flavonoids

Flavonoids are the second largest group of phenolic compounds in the five pear varieties. The subclass of flavonoids which were detected in pears include anthocyanins, dihydrochalcones, dihydroflavonols, flavanols, flavanones, flavones, flavonol and isoflavonoids. The flavanols and flavonols are the dominant subclasses among them with 7 of each group of compounds being detected.

##### Anthocyanins, Dihydrochalcones and Dihydroflavonols

Only 2 anthocyanins, 2 dihydrochalcones and 2 dihydroflavonols were detected in five pear varieties and both compounds **31** and **32** were detected in the positive ionization mode (Table 3). Compound **33** was identified as 3-hydroxyphloretin 2’-*O*-glucoside with the precursor ion [M − H] ^−^ at *m/z* 451.1236 and further confirmed by the MS^2^ experiment which shows a loss of glucoside (162 Da) at *m/z* 289 and phloretin aglycon at *m/z* 273 [60]. In addition, compound 35 was characterized as dihydroquercetin with the precursor ion at *m/z* 303.0501 in the negative ESI^−^ mode. Dihydroquercetin was also identified according to the MS^2^ fragmentation which exhibited the peaks at *m/z* 285, *m/z* 275 and *m/z* 151 by the loss of H_2_O, CO and 152 Da loss by RDA cleavage [61].

According to Raja et al. [62], dihydroquercetin is one of the highly specific polyphenols. They have identified this compound in two pear varieties, which are *conference* and *Alejandrina*. Previously, CHUNG [63] have identified petunidin 3-*O*-(6’’-acetyl-glucoside) in Highbush blueberry fruit with the MS value *m/z* at 521 and MS^2^ value *m/z* at 317, this result is same to our research. 3-Hydroxyphloretin 2’-*O*-glucoside in the peel of apples was also quantified in the previous study by HPLC analysis [64,65]. In addition, another dihydrochalcone, 3-hydroxyphloretin 2’-*O*-xylosyl-glucoside, was also identified in apple pomace by Alvarez Arraibi [66].

##### Flavanols

Only two (compounds **37** and **41**) out of seven flavanols were detected in more than a single pear variety. Compound **41** was tentatively characterized as (+)-catechin in the negative ESI^-^ mode at *m/z* 289.0712 and further confirmed by the MS^2^ experiment which displayed a characteristic loss of CO_2_ (44 Da), flavonoid a ring (84 Da) and flavonoid a ring (110 Da) at *m/z* 245, *m/z* 205 and *m/z* 179, respectively [37]. While compound **39** only presenting in Josephine de Malines in the negative ESI^-^ mode was proposed as procyanidin dimer B1 based on the [M − H]^−^ *m/z* at 577.1318. In the MS^2^ spectra, procyanidin dimer B1 showed the fragments at *m/z* 451 due to a loss of phloroglucinol (126 Da) [67].

Previously, Brahem et al. [7] detected the phenolic compounds in 19 pear varieties and characterized (+)-catechin in the flesh of four pear varieties and the peel of 15 pear varieties. In addition, the appearance of procyanidin dimer B1 and procyanidin trimer C1 in pear varieties were identified by de Pascual-Teresa et al. [68]. In the previous study, Yuzuak et al. [69] have confirmed the presence of (+)-gallocatechin in berries of Two Muscadine grape hybrids by applying HPLC-QTOF-MS/MS method. But Arts et al. [70] indicate that there was no (+)-gallocatechin in the peel and pulp of pears. The difference may result from the different pear varieties and the variance of the extraction method. In addition, (+)-catechin 3-*O*-gallate and 3’-*O*-methylcatechin were the first time identified in European pears to our best knowledge.

##### Flavonols

Four out of seven of flavonols were detected in only one pear varieties, which include compounds **50**, **51**, **52**, **53** (Table 3). Only compound **55** was detected in both negative and positive ionization modes. Two myricetin derivatives, which are myricetin 3-*O*-galactoside (compound **50**) and myricetin 3-*O*-rhamnoside (compound **56**), were characterized with the precursor ion [M − H] ^−^ at *m/z* 479.081 and *m/z* 463.0882 in the negative ESI^-^ mode. In the MS^2^ experiment, both compound **50** and compound **56** exhibited the same product ion at *m/z* 317 after the loss of glucoside (162 Da) and rhamnoside (146 Da) [71,72]. Compound **54** was identified as kaempferol 3,7-*O*-diglucoside with the precursor ion [M − H] ^−^ at *m/z* 609.1453 in the negative ionization mode and further confirmed by the MS^2^ experiment which displayed a characteristic loss of glucoside (162 Da) and two glucoside (324 Da) [73].

Previously, Dairpoosh [65] have characterized myricetin 3-*O*-rhamnoside in pear peel. In addition, myricetin 3-*O*-galactoside were characterized from grape wine by LC-MS method [74]. Barbosa et al. [75] reported that myricetin 3-*O*-rutinoside in *Chrysobalanus icaco* L. was detected in the negative mode with the quasi-molecular ion [M − H] ^−^ at *m/z* 625 and the fragment ion at *m/z* 317 corresponding to the loss of rhamnose unit. They showed slightly different results from our work, which might be due to the difference in mass spectrophotometric methods and techniques applied.

##### Flavanones, Flavones and Isoflavonoids

In total, 2 flavanones, 4 flavones and 3 isoflavonoids were detected in pear samples with five out of nine being found in only one pear variety (Table 3). Compound **58** was characterized as 3’,4’,7-trihydroxyisoflavanone which have the precursor ion [M − H] ^−^ at *m/z* 271.0612 in the positive ionization mode. The identity of 3’,4’,7-trihydroxyisoflavanone was proven by its MS/MS fragments at *m/z* 177, *m/z* 151 and *m/z* 119, corresponding to the loss of C_6_H_6_O (94 Da), C_8_H_8_O (120 Da) and C_7_H_4_O_4_ (152 Da), respectively [76]. Compound **46** was detected in the negative ESI^−^ mode and tentatively characterized as rhoifolin based on the precursor ion [M − H] ^−^ at *m/z* 577.1538. In the MS^2^ experiment, rhoifolin was identified based on the product ion at *m/z* 413 and *m/z* 269, produced by loss of rhamnose moiety and H_2_O (164 Da) and rhamnose moiety and glucose moiety (308 Da), respectively [76].

There have been very few studies focusing on the isoflavonoids in pears, although the isoflavonoid content of other fruits have been reported. Peng et al. [10] characterized 3’-hydroxydaidzein with [M + H]^+^ at *m/z* 271 in mango peel while 2-dehydro-*O*-desmethylangolensin has been identified in palm fruits and reported by Ma et al. [77]. Although no research has confirmed the presence of rhoifolin in pears, the presence of these compound in Pummelo juice and Mandarin juice has been reported by Nogata et al. [78]. Additionally, 6-hydroxyluteolin 7-*O*-rhamnoside can be found in Mexican oregano [79].

#### 3.4.3. Other Phenolic Compounds

Thirteen other polyphenols were also detected in the pear samples, which include 1 curcuminoids, 1 furanocoumarin, 2 hydroxybenzaldehydes, 2 hydroxybenzoketones, 1 hydroxycoumarins, 1 phenolic terpene, 2 tyrosols and 3 other phenolic compounds (Table 3). Only two of these compounds (Compounds **63** and **66**), were observed in both negative and positive ionization modes. Compound **64** and was found in in all pear varieties except Winter Nelis and was identified as 4-hydroxybenzaldehyde having the precursor ion [M − H] ^−^ at *m/z* 121.0298 in the negative ionization mode and further confirmed by the MS^2^ experiment which displayed a characteristic loss of CO_2_ (44Da) [80]. Compound **68** was tentatively characterized in the negative ionization mode as arbutin based on the precursor ion [M − H] ^−^ at *m/z* 271.0828 and further identified by MS/MS spectra which showed a loss of glucoside (162 Da) [81]. In addition, carnosic acid (compound **71**) was observed in Packham’s Triumph with the precursor ion [M − H] ^−^ at *m/z* 331.1905. In the MS^2^ spectra of carnosic acid, the product ion at *m/z* 287 and *m/z* 269 were due to the loss CO_2_ and further loss of H_2_O from the parent ion [82].

Arbutin is an important phenolic compound with antibiotic properties and has been confirmed as the dominant phenolic compounds in 8 varieties of pears by Öztürk et al. [47] and Li et al. [83]. Some of the other compounds were reported in other plants but not pears. Pistelli et al. [84] have identified the presence of demethoxycurcumin in *Curcuma longa* by LC-DAD-ESI-MS method. While the presence of isopimpinellin have also been confirmed by Dehghan et al. [85] in *Heracleum persicum*. In addition, Welke et al. [86] used comprehensive two-dimensional gas chromatography to identify *p*-anisaldehyde in the Chardonnay wine. In addition, both lithospermic acid salvianolic acid B were identified in Chinese Wild *Salvia miltiorrhiza* based on UPLC-QqQ-MS method by Zhang et al. [87]. In addition, although carnosic acid was not identified in pears to our best knowledge, but it has been reported widely distributed in the plant species of the Lamiaceae family [88].

### 3.5. HPLC Analysis

In the present work, the quantification of phenolic compounds was based on comparing retention time with HPLC grade reference standards. In total, 10 phenolic compounds were quantified through HPLC-PDA, including five targeted phenolic acids (gallic acid, protocatechuic acid, *p*-hydroxybenzoic acid, chlorogenic acid and caffeic acid) and five flavonoids (catechin, epicatechin, epicatechin gallate, quercetin and kaempferol) (Table 4).

Beurre Bosc has the highest phenolic acid contents while Winter Nelis and Rico have lower phenolic acids contents (Table 4) which is consistent with the other estimations of TPC in the present study. Among the five targeted phenolic acids, protocatechuic acid was only detected in Beurre Bosc, Josephine de Malines and Packham’s Triumph. In addition, a small amount of *p*-hydroxybenzoic acid was detected in five pear varieties, which ranged from 0.95 ± 0.05 mg/g to 3.14 ± 0.15 mg/g. They were abundant in Packham’s Triumph and less abundant in Josephine de Malines and Rico, respectively. Chlorogenic acid is the dominant phenolic acids among the five selected phenolic acids. It is abundant in all five pear varieties except Rico. Previously, both Öztürk et al. [47] and Liaudanskas et al. [89] have reported that chlorogenic acid is the dominant phenolic compounds in several Lithuania and Sinop grown pear varieties, including Conference, Concordia, Grabova and Patten. Further, protocatechuic acid was also previously quantified by Truong et al. [90] through HPLC analysis in the Asian grown pear varieties (*Pyrus* spp.). In addition, Tanrıöven and Ekşi [91] have quantitated chlorogenic acid and caffeic acid in pear juice using HPLC method from several pear varieties, for instance, Williams, Santa Maria and Starkrimson. In the previous study, both Li et al. [21] and Li et al. [92] have quantitated the gallic acid in different pear varieties growing in China, which includes *P. bretschneideri, P. pyrifolia, P. pyrifolia Nakai* and *Pyrus* sp. nr. *Communis*.

Regarding flavonoids, catechin is the major flavonoids in the five pear varieties except for Packham’s Triumph. It is also the dominant flavonoids among the five selected flavonoids, this is consistent with the conclusion from Öztürk et al. [89]. In a small amount, epicatechin gallate was only detected in three pear varieties, which are Beurre Bosc, Josephine de Malines and Rico. Epicatechin is high in Beurre Bosc but less abundant in Winter Nelis. In addition, quercetin is abundant in Josephine de Malines and low in Packham’s Triumph and Rico. In the previous study, Brahem et al. [7] also quantified the epicatechin in the flesh and peel of 16 European grown pear varieties (such as Rochas, William rouge and William vert) and found that the concentration is higher in the peel than flesh. However, Arts et al. [70] quantified that there is no epicatechin gallate detected in 2 Netherlands grown pear varieties, including Conference and Doyenne du Comice. This may be due to the difference in pear varieties, growing regions and extraction solvent.

## 4. Conclusions

Based on these data, Beurre Bosc has the highest TPC, TTC and DPPH concentrations while Josephine de Malines was high in TFC, ABTS, FRAP and TAC values. Winter Nelis was consistently low across the antioxidant assays except for TAC. The present work successfully employed LC-ESI-QTOF-MS/MS analysis to sperate and identify the phenolic profile in the pulp of five Australian grown pear varieties. A total of 73 phenolic compounds were detected in the five Australian grown pear varieties studied. The present work also used HPLC to quantify the phenolic compounds in pears and a total of five phenolic acids and five flavonoids were quantified. Chlorogenic acid and catechin are the dominant phenolic compounds in pears. This project provides comprehensive qualitative and quantitative information about the phenolic compounds present in Australian grown pears. These data may be useful for developing strategies and products in the nutraceutical, pharmaceutical and food industries.

## Figures and Tables

**Table 1:** Antioxidant potentials of five Australian grown pear varieties.

Antioxidant Assays	Beurre Bosc	Josephinede Malines	Packham’s Triumph	Winter Nelis	Rico
TPC (mg GAE/g)	3.14 ± 0.02 ^a^	2.75 ± 0.03 ^b^	2.19 ± 0.06 ^d^	1.89 ± 0.03 ^e^	2.45 ± 0.09 ^c^
TFC (mg QE/g)	1.04 ± 0.07 ^b^	1.53 ± 0.09 ^a^	0.94 ± 0.07 ^c^	0.57 ± 0.05 ^e^	0.74 ± 0.07 ^d^
TTC (mg CE/g)	1.43 ± 0.04 ^a^	1.01 ± 0.04 ^c^	0.81 ± 0.03 ^d^	0.72 ± 0.09 ^d^	1.10 ± 0.03 ^b^
DPPH (mg AAE/g)	5.72 ± 0.11 ^a^	4.78 ± 0.06 ^b^	4.12 ± 0.09 ^c^	3.25 ± 0.03 ^d^	4.72 ± 0.06 ^b^
FRAP (mg AAE/g)	3.93 ± 0.04 ^b^	4.37 ± 0.04 ^a^	3.14 ± 0.07 ^c^	2.15 ± 0.07 ^e^	2.94 ± 0.01 ^d^
ABTS (mg AAE/g)	4.41 ± 0.07 ^a^	4.44 ± 0.01 ^a^	3.97 ± 0.10 ^b^	2.83 ± 0.06 ^d^	3.19 ± 0.04 ^c^
TAC (mg AAE/g)	3.92 ± 0.04 ^c^	5.29 ± 0.09 ^a^	4.85 ± 0.03 ^b^	3.94 ± 0.01 ^c^	2.87 ± 0.09 ^d^

^a,b,c,d,e^ indicate the means in a row with significant difference (*p* < 0.05) using a one-way analysis of variance (ANOVA) and Tukey’s test. TPC, total phenolic content; TFC, total flavonoid content; TTC, total tannin content; DPPH, 2,2′-diphenyl-1-picrylhydrazyl assay; FRAP, ferric reducing antioxidant power assay; ABTS, 2,2′-azino-bis-3-ethylbenzothiazoline-6-sulfonic acid assay; TAC, total antioxidant capacity; GAE, gallic acid equivalents; QE, quercetin equivalents; CE, catechin equivalents; AAE, ascorbic acid equivalents.

**Table 2:** Pearson’s correlation between antioxidant capacity by different antioxidant assays.

Variables	TPC	TFC	TTC	DPPH	FRAP	ABTS
TFC	0.634					
TTC	0.934 *	0.322				
DPPH	0.973 **	0.529	0.958 **			
FRAP	0.854	0.939 **	0.619	0.785		
ABTS	0.791	0.869 *	0.555	0.731	0.940 **	
TAC	0.063	0.716	−0.279	−0.072	0.510	0.621

** Significant correlation with *p* ≤ 0.01; * Significant correlation with *p* ≤ 0.05.

**Table 3:** Characterization of phenolic compounds in different pear samples by liquid chromatography coupled with electrospray-ionization quadrupole time-of-flight mass spectrometry (LC-ESI-QTOF-MS/MS).

No.	Molecular Formula	Proposed Compounds	RT (min)	Ionization (ESI^+^/ESI^−^)	Molecular Weight	Theoretical (*m/z*)	Observed (*m/z*)	Error (ppm)	MS^2^ Production	Pears
**Phenolic acid**										
	**Hydroxybenzoic Acids**									
1	C_8_H_8_O_7_S	Vanillic acid 4-sulfate	5.122	[M − H] ^−^	247.9991	246.9918	246.9915	−1.2	167	BB
2	C_13_H_16_O_10_	Gallic acid 4-*O*-glucoside	6.731	[M − H] ^−^	332.0743	331.067	331.0675	1.5	169, 125	* RI, BB, JM
3	C_7_H_6_O_5_	Gallic acid	6.878	[M − H] ^−^	170.0215	169.0142	169.0145	1.8	125	BB
4	C_13_H_16_O_9_	Protocatechuic acid 4-*O*-glucoside	7.524	[M − H] ^−^	316.0794	315.0721	315.0718	−1.0	153	* BB, RI, JM, PT
5	C_7_H_6_O_3_	2-Hydroxybenzoic acid	11.185	[M − H] ^−^	138.0317	137.0244	137.025	4.4	93	* BB, JM, RI, PT
6	C_13_H_16_O_8_	4-Hydroxybenzoic acid 4-*O*-glucoside	11.218	[M − H] ^−^	300.0845	299.0772	299.0759	−4.3	255, 137	* BB, WN
7	C_14_H_10_O_9_	Gallic acid 3-*O*-gallate	17.066	[M − H] ^−^	322.0325	321.0252	321.0239	−4.0	169	BB
8	C_7_H_6_O_4_	2,3-Dihydroxybenzoic acid	24.242	[M − H] ^−^	154.0266	153.0193	153.0192	−0.7	109	* RI, JM
	**Hydroxycinnamic Acids**									
9	C_9_H_8_O_3_	*m*-Coumaric acid	5.207	[M − H] ^−^	164.0473	163.04	163.0397	−1.8	119	* RI, JM, PT
10	C_9_H_8_O_2_	Cinnamic acid	9.219	** [M − H] ^−^	148.0524	147.0451	147.0461	4.8	103	* WN, RI, PT, BB
11	C_14_H_14_O_9_	Feruloyl tartaric acid	10.506	[M − H] ^−^	326.0638	325.0565	325.0567	0.6	193, 149	BB
12	C_9_H_8_O_4_	Caffeic acid	12.932	** [M − H] ^−^	180.0423	179.035	179.0346	−2.2	143, 133	* JM, BB, RI
13	C_15_H_16_O_10_	Caffeic acid 3-*O*-glucuronide	13.308	[M − H] ^−^	356.0743	355.067	355.0672	0.6	179	RI
14	C_15_H_18_O_8_	*p*-Coumaric acid 4-*O*-glucoside	14.962	[M − H] ^−^	326.1002	325.0929	325.0911	−5.5	163	BB
15	C_15_H_18_O_9_	Caffeoyl glucose	19.343	[M − H] ^−^	342.0951	341.0878	341.0865	−3.8	179, 161	* JM, PT
16	C_16_H_18_O_9_	3-Caffeoylquinic acid	24.793	[M − H] ^−^	354.0951	353.0878	353.0865	−3.7	253, 190, 144	JM
17	C_16_H_18_O_10_	Ferulic acid 4-*O*-glucuronide	26.748	[M − H] ^−^	370.09	369.0827	369.0838	3.0	193	JM
18	C_16_H_18_O_8_	3-*p*-Coumaroylquinic acid	27.825	[M − H] ^−^	338.1002	337.0929	337.0918	−3.3	265, 173, 162	JM
19	C_13_H_12_O_8_	*p*-Coumaroyl tartaric acid	28.947	** [M − H] ^−^	296.0532	295.0459	295.0457	−0.7	115	* RI, BB
20	C_17_H_20_O_9_	3-Feruloylquinic acid	29.432	** [M − H] ^−^	368.1107	367.1034	367.1028	−1.6	298, 288, 192, 191	* JM, BB, WN
21	C_16_H_20_O_9_	Ferulic acid 4-*O*-glucoside	33.867	[M − H] ^−^	356.1107	355.1034	355.1039	1.4	193, 178, 149, 134	RI
22	C_10_H_10_O_4_	Ferulic acid	38.378	[M − H] ^−^	194.0579	193.0506	193.0499	−3.6	178, 149, 134	JM
23	C_18_H_16_O_8_	Rosmarinic acid	39.746	[M − H] ^−^	360.0845	359.0772	359.0773	0.3	179	BB
24	C_25_H_24_O_12_	1,5-Dicaffeoylquinic acid	45.17	[M − H] ^−^	516.1268	515.1195	515.1176	−3.7	353, 335, 191, 179	JM
25	C_15_H_18_O_7_	Cinnamoyl glucose	60.985	[M − H] ^−^	310.1053	309.098	309.0965	−4.9	147, 131, 103	BB
	**Hydroxyphenylacetic Acids**									
26	C_8_H_8_O_4_	3,4-Dihydroxyphenylacetic acid	10.011	[M − H] ^−^	168.0423	167.035	167.0349	−0.6	149, 123	* RI, BB, WN, JM
27	C_8_H_8_O_3_	2-Hydroxy-2-phenylacetic acid	10.821	[M − H] ^−^	152.0473	151.04	151.0405	3.3	136, 92	BB
	**Hydroxyphenylpropanoic Acids**									
28	C_9_H_10_O_4_	3-Hydroxy-3-(3-hydroxyphenyl) propionic acid	14.73	[M − H] ^−^	182.0579	181.0506	181.0504	−1.1	163, 135, 119	BB
29	C_15_H_18_O_10_	Dihydrocaffeic acid 3-*O*-glucuronide	20.796	[M − H] ^−^	358.09	357.0827	357.0828	0.3	181	* RI, BB
30	C_16_H_20_O_10_	Dihydroferulic acid 4-*O*-glucuronide	29.117	[M − H] ^−^	372.1056	371.0983	371.0975	−2.2	195	JM
**Flavonoid**										
	**Anthocyanins**									
31	C_24_H_25_O_13_	Petunidin 3-*O*-(6’’-acetyl-glucoside)	27.386	[M + H]^+^	521.1295	522.1368	522.1358	−1.9	317	WN
32	C_43_H_49_O_24_	Cyanidin 3-*O*-(2-*O*-(6-*O*-(*E*)-caffeoyl-*D* glucoside)-*D*-glucoside)-5-*O*-*D*-glucoside	40.107	[M + H]^+^	949.2614	950.2687	950.2673	−1.5	787, 463, 301	BB
	**Dihydrochalcones**									
33	C_21_H_24_O_11_	3-Hydroxyphloretin 2’-*O*-glucoside	13.819	[M − H] ^−^	452.1319	451.1246	451.1236	−2.2	289, 273	BB
34	C_26_H_32_O_15_	3-Hydroxyphloretin 2’-*O*-xylosyl-glucoside	36.847	[M − H] ^−^	584.1741	583.1668	583.1677	1.5	289	* BB, RI
	**Dihydroflavonols**									
35	C_15_H_12_O_7_	Dihydroquercetin	11.732	[M − H] ^−^	304.0583	303.051	303.0501	−3.0	285, 275, 151	BB
36	C_21_H_22_O_12_	Dihydromyricetin 3-*O*-rhamnoside	34.071	[M − H] ^−^	466.1111	465.1038	465.1044	1.3	301	JM
	**Flavanols**									
37	C_15_H_14_O_7_	(+)-Gallocatechin	4.676	[M − H] ^−^	306.074	305.0667	305.0676	3.0	261, 219	* PT, BB
38	C_22_H_24_O_13_	4’-*O*-Methyl-(-)-epigallocatechin 7-*O*-glucuronide	6.911	[M − H] ^−^	496.1217	495.1144	495.1153	1.8	451, 313	BB
39	C_30_H_26_O_12_	Procyanidin dimer B1	23.22	[M − H] ^−^	578.1424	577.1351	577.1318	−5.7	451	JM
40	C_16_H_16_O_6_	3’-*O*-Methylcatechin	24.124	[M − H] ^−^	304.0947	303.0874	303.0878	1.3	271, 163	BB
41	C_15_H_14_O_6_	(+)-Catechin	26.351	[M − H] ^−^	290.079	289.0717	289.0712	−1.7	245, 205, 179	* JM, BB
42	C_45_H_38_O_18_	Procyanidin trimer C1	28.687	[M − H] ^−^	866.2058	865.1985	865.1941	−5.1	739, 713, 695	JM
43	C_22_H_18_O_10_	(+)-Catechin 3-*O*-gallate	36.333	[M − H] ^−^	442.09	441.0827	441.0825	−0.5	289, 169, 125	BB
	**Flavanones**									
44	C_28_H_30_O_18_	Hesperetin 3’,7-*O*-diglucuronide	9.315	[M − H] ^−^	654.1432	653.1359	653.1362	0.5	477, 301, 286, 242	RI
45	C_22_H_22_O_12_	Hesperetin 3’-*O*-glucuronide	47.368	[M − H] ^−^	478.1111	477.1038	477.1022	−3.4	301, 286, 257, 242	PT
	**Flavones**									
46	C_27_H_30_O_14_	Rhoifolin	27.229	[M − H] ^−^	578.1636	577.1563	577.1538	−4.3	413, 269	JM
47	C_27_H_30_O_15_	Apigenin 6,8-di-C-glucoside	42.901	** [M − H] ^−^	594.1585	593.1512	593.1485	−4.6	503, 473	* JM, WN
48	C_21_H_20_O_11_	6-Hydroxyluteolin 7-*O*-rhamnoside	46.341	** [M − H] ^−^	448.1006	447.0933	447.0915	−4.0	301	* PT, JM, WN, BB
49	C_15_H_10_O_4_	7,4’-Dihydroxyflavone	82.529	[M + H]^+^	254.0579	255.0652	255.0646	−2.4	227, 199, 171	* RI, BB
	**Flavonols**									
50	C_21_H_20_O1_3_	Myricetin 3-*O*-galactoside	19.288	[M − H] ^−^	480.0904	479.0831	479.081	−4.4	317	PT
51	C_27_H_30_O_17_	Myricetin 3-*O*-rutinoside	31.52	[M − H] ^−^	626.1483	625.141	625.1386	−3.8	301	JM
52	C_43_H_48_O_24_	Spinacetin 3-*O*-(2’’-*p*-coumaroylglucosyl) (1->6)- [apiosyl (1->2)]-glucoside	33.242	[M − H] ^−^	948.2536	947.2463	947.2456	−0.7	741, 609, 301	JM
53	C_33_H_40_O_19_	Kaempferol 3-*O*-(2’’-rhamnosyl-galactoside) 7-*O*-rhamnoside	35.077	[M − H] ^−^	740.2164	739.2091	739.2091	0.0	593, 447, 285	RI
54	C_27_H_30_O_16_	Kaempferol 3,7-*O*-diglucoside	37.384	[M − H] ^−^	610.1534	609.1461	609.1453	−1.3	447, 285	* JM, RI
55	C_33_H_40_O_20_	Kaempferol 3-*O*-glucosyl-rhamnosyl-galactoside	40.184	** [M − H] ^−^	756.2113	755.204	755.2047	0.9	285	* JM, WN, RI
56	C_21_H_20_O_12_	Myricetin 3-*O*-rhamnoside	40.234	[M − H] ^−^	464.0955	463.0882	463.0882	0.0	317	* JM, PT
	**Isoflavonoids**									
57	C_15_H_12_O_4_	2-Dehydro-*O*-desmethylangolensin	77.899	[M − H] ^−^	256.0736	255.0663	255.0678	5.9	135, 119	BB
58	C_15_H_12_O_5_	3’,4’,7-Trihydroxyisoflavanone	78.287	[M − H] ^−^	272.0685	271.0612	271.0612	0.0	177, 151, 119, 107	* JM, BB
59	C_15_H_10_O_5_	3’-Hydroxydaidzein	81.816	[M + H]^+^	270.0528	271.0601	271.0591	−3.7	253, 241, 225	RI
**Stilbenes**										
60	C_20_H_22_O_8_	Resveratrol 5-*O*-glucoside	38.063	[M − H] ^−^	390.1315	389.1242	389.1245	0.8	227	JM
**Other Polyphenols**										
	**Curcuminoids**									
61	C_20_H_18_O_5_	Demethoxycurcumin	81.976	[M − H] ^−^	338.1154	337.1081	337.108	−0.3	217	PT
	**Furanocoumarins**									
62	C_13_H_10_O_5_	Isopimpinellin	4.478	[M + H]^+^	246.0528	247.0601	247.0605	1.6	232, 217, 205, 203	PT
	**Hydroxybenzaldehydes**									
63	C_8_H_8_O_2_	*p*-Anisaldehyde	30.338	**[M + H]^+^	136.0524	137.0597	137.0599	1.5	122, 109	* JM, BB
64	C_7_H_6_O_2_	4-Hydroxybenzaldehyde	44.756	[M − H] ^−^	122.0368	121.0295	121.0298	2.5	77	* JM, PT, BB, RI
	**Hydroxybenzoketones**									
65	C_9_H_10_O_7_S	2-Hydroxy-4-methoxyacetophenone 5-sulfate	9.446	[M − H] ^−^	262.0147	261.0074	261.0067	−2.7	181,97	BB
66	C_10_H_12_O_5_	2,3-Dihydroxy-1-guaiacylpropanone	21.442	**[M − H] ^−^	212.0685	211.0612	211.0605	−3.3	167, 123, 105, 93	* RI, JM
	**Hydroxycoumarins**									
67	C_10_H_8_O_4_	Scopoletin	43.298	[M − H] ^−^	192.0423	191.035	191.0361	5.8	176	JM
	**Other Polyphenols**									
68	C_12_H_16_O_7_	Arbutin	5.129	[M − H] ^−^	272.0896	271.0823	271.0828	1.8	109	JM
69	C_27_H_22_O_12_	Lithospermic acid	5.834	[M − H] ^−^	538.1111	537.1038	537.1037	−0.2	493, 339, 295	BB
70	C_36_H_30_O_16_	Salvianolic acid B	28.598	[M − H] ^−^	718.1534	717.1461	717.1436	−3.5	519, 339, 321, 295	PT
	**Phenolic Terpenes**									
71	C_20_H_28_O_4_	Carnosic acid	80.419	[M − H] ^−^	332.1988	331.1915	331.1905	−3.0	287, 269	PT
	**Tyrosols**									
72	C_10_H_12_O_4_	3,4-DHPEA-AC	8.172	[M − H] ^−^	196.0736	195.0663	195.0666	1.5	135	* RI, WN
73	C_14_H_20_O_8_	Hydroxytyrosol 4-*O*-glucoside	9.777	[M − H] ^−^	316.1158	315.1085	315.1076	−2.9	153, 123	BB

** Compounds were detected in both negative [M − H] ^−^ and positive [M+H]^+^ mode of ionization while only single mode data was presented. Pear samples mentioned in abbreviations are Packham’s Triumph “PT”, Josephine de Malines “JM”, Beurre Bosc “BB’’, Winter Nelis “WN” and Rico “RI”.

**Table 4:** Quantification of phenolic compounds in pears by using HPLC-PDA.

No.	Compound Name	RT (min)	Beurre Bosc (mg/g)	Josephine de Malines (mg/g)	Packham’s Triumph (mg/g)	Winter Nelis (mg/g)	Rico (mg/g)	Polyphenol Classes
1	Gallic acid	6.836	5.68 ± 0.34 ^a^	3.25 ± 0.16 ^b^	0.25 ± 0.02 ^d^	1.28 ± 0.07 ^c^	2.43 ± 0.21 ^b^	Phenolic acid
2	Protocatechuic acid	12.569	3.54 ± 0.31 ^a^	1.27 ± 0.11 ^c^	2.41 ± 0.12 ^b^	-	-	Phenolic acid
3	*p*-Hydroxybenzoic acid	20.24	2.15 ± 0.17 ^b^	1.64 ± 0.11 ^c^	3.14 ± 0.15 ^a^	2.14 ± 0.10 ^b^	0.95 ± 0.05 ^d^	Phenolic acid
4	Chlorogenic acid	20.579	17.58 ± 0.88 ^a^	9.78 ± 0.78 ^d^	12.35 ± 0.99 ^c^	14.51 ± 0.87 ^b^	1.53 ± 0.13 ^e^	Phenolic acid
5	Caffeic acid	25.001	3.58 ± 0.21 ^b^	1.85 ± 0.11 ^c^	2.48 ± 0.14 ^c^	0.98 ± 0.09 ^d^	4.57 ± 0.36 ^a^	Phenolic acid
6	Catechin	19.704	14.89 ± 0.89 ^b^	17.45 ± 1.39 ^a^	4.59 ± 0.41 ^e^	9.45 ± 0.75 ^d^	11.25 ± 1.01 ^c^	Flavonoid
7	Epicatechin	24.961	6.98 ± 0.49 ^a^	3.64 ± 0.15 ^b^	2.37 ± 0.19 ^c^	1.58 ± 0.14 ^d^	2.31 ± 0.20 ^c^	Flavonoid
8	Epicatechin gallate	38.015	2.31 ± 0.11 ^a^	1.89 ± 0.13 ^a^	-	-	1.25 ± 0.08 ^b^	Flavonoid
9	Quercetin	70.098	6.38 ± 0.44 ^b^	14.57 ± 1.01 ^a^	3.28 ± 0.16 ^c^	5.49 ± 0.44 ^b^	4.58 ± 0.23 ^b^	Flavonoid
10	Kaempferol	80.347	3.37 ± 0.17 ^c^	4.58 ± 0.41 ^b^	8.59 ± 0.60 ^a^	3.27 ± 0.16 ^c^	1.28 ± 0.10 ^d^	Flavonoid

All data are the mean ± SD of three replicates. Means followed by different letters (^a,b,c,d,e^) within the same column are significantly different (*p* < 0.05) of each other. Data of Packham’s Triumph, Josephine de Malines, Beurre Bosc, Winter Nelis and Rico are reported on a dry weight basis.

## Data Availability

Not applicable.

## Supplementary Materials

The following are available online at https://www.mdpi.com/2076-3921/10/2/151/s1, Figure S1: LC-ESI-QTOF-MS/MS basic peak chromatograph (BPC) for characterization of phenolic compounds of Australian grown pear varieties. Figure S2. Extracted ion chromatogram of pear sample and their mass spectrum.

## References

[1] Kolniak-Ostek J. (2016). Chemical composition and antioxidant capacity of different anatomical parts of pear (Pyrus communis L.). Food Chem..

[2] Silva G.J., Souza T.M., Barbieri R.L., De Oliveira A.C. (2014). Origin, Domestication, and Dispersing of Pear (Pyrus spp.). Adv. Agric..

[3] Jun-feng G., Xiao-dong D. (2017). Present situation and future of pear processing technology in china. Storage Process.

[4] Yim S., Nam S. (2016). Physiochemical, nutritional and functional characterization of 10 different pear cultivars (pyrus spp.). J. Appl. Bot. Food Qual..

[5] Borges G., Mullen W., Crozier A. (2010). Comparison of the polyphenolic composition and antioxidant activity of European commercial fruit juices. Food Funct..

[6] Katz I.H., Nagar E.E., Okun Z., Shpigelman A. (2020). The Link between Polyphenol Structure, Antioxidant Capacity and Shelf-Life Stability in the Presence of Fructose and Ascorbic Acid. Molecules.

[7] Brahem M., Renard C.M., Eder S., Loonis M., Ouni R., Mars M., Le Bourvellec C. (2017). Characterization and quantification of fruit phenolic compounds of European and Tunisian pear cultivars. Food Res. Int..

[8] Amorati R., Valgimigli L. (2015). Advantages and limitations of common testing methods for antioxidants. Free Radic. Res..

[9] Jie Z., Hongbao W., Jiajun K., Xiaofei S., Shutian T. (2017). Purification and antioxidant activity of polyphenols from young pear fruits. Food Sci. China.

[10] Peng D., Zahid H.F., Ajlouni S., Dunshea F.R., Suleria H.A.R. (2019). LC-ESI-QTOF/MS Profiling of Australian Mango Peel By-Product Polyphenols and Their Potential Antioxidant Activities. Process.

[11] Tang J., Dunshea F.R., Suleria H.A.R. (2019). LC-ESI-QTOF/MS Characterization of Phenolic Compounds from Medicinal Plants (Hops and Juniper Berries) and Their Antioxidant Activity. Foods.

[12] Samsonowicz M., Regulska E., Karpowicz D., Leśniewska B. (2019). Antioxidant properties of coffee substitutes rich in polyphenols and minerals. Food Chem..

[13] Stavrou I.J., Christou A., Kapnissi-Christodoulou C.P. (2018). Polyphenols in carobs: A review on their composition, antioxidant capacity and cytotoxic effects, and health impact. Food Chem..

[14] Sogi D.S., Siddiq M., Greiby I., Dolan K.D. (2013). Total phenolics, antioxidant activity, and functional properties of ‘Tommy Atkins’ mango peel and kernel as affected by drying methods. Food Chem..

[15] Subbiah V., Zhong B., Nawaz M.A., Barrow C.J., Dunshea F.R., Suleria H.A.R. (2021). Screening of Phenolic Compounds in Australian Grown Berries by LC-ESI-QTOF-MS/MS and Determination of Their Antioxidant Potential. Antioxidants.

[16] Suleria H.A.R., Barrow C.J., Dunshea F.R. (2020). Screening and Characterization of Phenolic Compounds and Their Antioxidant Capacity in Different Fruit Peels. Foods.

[17] Zhong B., Robinson N.A., Warner R., Barrow C.J., Dunshea F.R., Suleria H.A.R. (2020). LC-ESI-QTOF-MS/MS Characterization of Seaweed Phenolics and Their Antioxidant Potential. Mar. Drugs.

[18] Erbil N., Murathan Z.T., Arslan M., Ilcim A., Sayin B. (2018). Antimicrobial, Antioxidant, and Antimutagenic Activities of Five Turkish Pear Cultivars. Erwerbs-Obstbau.

[19] Manzoor M., Anwar F., Bhatti I.A., Jamil A. (2013). Variation of phenolics and antioxidant activity between peel and pulp parts of pear (pyrus communis L.) fruit. Pak. J. Bot..

[20] Azzini E., Maiani G., Durazzo A., Foddai M.S., Intorre F., Venneria E., Forte V., Lucchetti S., Ambra R., Pastore G. (2019). Giovanni Varieties (Pyrus communis L.): Antioxidant Properties and Phytochemical Characteristics. Oxidative Med. Cell. Longev..

[21] Li X., Wang T., Zhou B., Gao W., Cao J., Huang L. (2014). Chemical composition and antioxidant and anti-inflammatory potential of peels and flesh from 10 different pear varieties (Pyrus spp.). Food Chem..

[22] Rawat P., Saroj N., Rawat P., Kumar P., Singh T.D., Pal M. (2015). Evaluation for total phenolic, total flavonoid and antioxidant activity of leaves and roots of pyrus pashia. Int. J. Med. Pharm. Res.

[23] Patricia V.M., Syaputri F.N., Tugon T.D.A., Mardhatillah A. (2020). Antioxidant Properties of Pyrus communis and Pyrus pyrifolia Peel Extracts. Borneo J. Pharm..

[24] Ma J.N., Wang S.L., Zhang K., Wu Z.G., Hattori M., Chen G.L., Ma C.M. (2012). Chemical components and antioxidant activity of the peels of commercial apple-shaped pear (fruit of pyrus pyrifolia cv. Pingguoli). J. Food Sci..

[25] Velmurugan C., Bhargava A. (2014). Total phenolic, flavonoids and tannin content of various extracts from pyrus communis fruit. Int. J. Pharm. Anal. Res..

[26] Nomura K., Takaoka M., Uematsu C., Ieguchi T., Katayama H. Pear (pyrus l.) genetic resources from northern japan: Evaluation of antioxidant capacity. Proceedings of the XII International Pear Symposium 1094.

[27] Galvis-Sánchez A.C., Gil-Izquierdo A., Gil M.I. (2003). Comparative study of six pear cultivars in terms of their phenolic and vitamin C contents and antioxidant capacity. J. Sci. Food Agric..

[28] Syrgiannidis G., Sotiropoulos T., Petridis A., Therios I. (2011). ‘Vergina’ pear. HortScience.

[29] Sotiropoulos T., Koutinas N., Giannakoula A. (2016). ‘Naoussa’ pear. HortScience.

[30] Gu C., Howell K., Dunshea F.R., Suleria H.A.R. (2019). LC-ESI-QTOF/MS Characterisation of Phenolic Acids and Flavonoids in Polyphenol-Rich Fruits and Vegetables and Their Potential Antioxidant Activities. Antioxidants.

[31] Jamuna K., Ramesh C., Srinivasa T., Raghu K. (2011). In vitro antioxidant studies in some common fruits. Int. J. Pharm. Pharm. Sci..

[32] Batista S., Guiné R., Barroca M.J., Gonçalves F., Pérez M.D., San José M., Ferreira D. (2005). Sun-dried pears: Phenolic compounds and antioxidant activity. 7° Encontro Química Aliment..

[33] Jamuna K., Ramesh C., Srinivasa T., Raghu K. (2011). Total antioxidant capacity in aqueous extracts of some common fruits. Int. J. Pharm. Sci. Res..

[34] Floegel A., Kim D.-O., Chung S.-J., Koo S.I., Chun O.K. (2011). Comparison of ABTS/DPPH assays to measure antioxidant capacity in popular antioxidant-rich US foods. J. Food Compos. Anal..

[35] Du G., Li M., Ma F., Liang D. (2009). Antioxidant capacity and the relationship with polyphenol and Vitamin C in Actinidia fruits. Food Chem..

[36] Luna-Guevara M.L., Luna-Guevara J.J., Hernández-Carranza P., Ruíz-Espinosa H., Ochoa-Velasco C.E. (2018). Phenolic compounds: A good choice against chronic degenerative diseases. Studies in Natural Products Chemistry.

[37] Escobar-Avello D., Lozano-Castellón J., Mardones C., Pérez A.J., Saéz V., Riquelme S., von Baer D., Vallverdú-Queralt A. (2019). Phenolic profile of grape canes: Novel compounds identified by lc-esi-ltq-orbitrap-ms. Molecules.

[38] Rajauria G., Foley B., Abu-Ghannam N. (2016). Identification and characterization of phenolic antioxidant compounds from brown Irish seaweed Himanthalia elongata using LC-DAD–ESI-MS/MS. Innov. Food Sci. Emerg. Technol..

[39] Chen S., Lu C., Zhao R. (2015). Identification and Quantitative Characterization of PSORI-CM01, a Chinese Medicine Formula for Psoriasis Therapy, by Liquid Chromatography Coupled with an LTQ Orbitrap Mass Spectrometer. Molecules.

[40] Catarino M.D., Silva A.M., Saraiva S.C., Sobral A.J., Cardoso S.M. (2018). Characterization of phenolic constituents and evaluation of antioxidant properties of leaves and stems of Eriocephalus africanus. Arab. J. Chem..

[41] Robertson G.L., Kermode W.J. (1981). Salicylic acid in fresh and canned fruit and vegetables. J. Sci. Food Agric..

[42] Pj B., Shibumon G., Sunny K., Cincy G. (2010). 2,3-dihydroxybenzoic acid: An effective antifungal agent isolated from flacourtia inermis fruit. Int. J. Pharm. Clin. Res..

[43] Wang J., Jia Z., Zhang Z., Wang Y., Liu X., Wang L., Lin R. (2017). Analysis of Chemical Constituents of Melastoma dodecandrum Lour. by UPLC-ESI-Q-Exactive Focus-MS/MS. Molecules.

[44] Lin H., Zhu H., Tan J., Wang H., Wang Z., Li P., Zhao C., Liu J. (2019). Comparative analysis of chemical constituents of moringa oleifera leaves from china and india by ultra-performance liquid chromatography coupled with quadrupole-time-of-flight mass spectrometry. Molecules.

[45] Lai K.-M., Cheng Y.-Y., Tsai T.-H. (2015). Integrated LC-MS/MS Analytical Systems and Physical Inspection for the Analysis of a Botanical Herbal Preparation. Molecules.

[46] Sun L., Tao S., Zhang S. (2019). Characterization and Quantification of Polyphenols and Triterpenoids in Thinned Young Fruits of Ten Pear Varieties by UPLC-Q TRAP-MS/MS. Molecules.

[47] Öztürk A., Demirsoy L., Demirsoy H., Asan A., Gül O. (2014). Phenolic compounds and chemical characteristics of pears (pyrus communis l.). Int. J. Food Prop..

[48] Simirgiotis M., Quispe C., Bórquez J., Areche C., Sepulveda B. (2016). Fast Detection of Phenolic Compounds in Extracts of Easter Pears (Pyrus communis) from the Atacama Desert by Ultrahigh-Performance Liquid Chromatography and Mass Spectrometry (UHPLC–Q/Orbitrap/MS/MS). Molecules.

[49] Hudina M., Stampar F., Orazem P., Petkovsek M.M., Veberic R. (2012). Phenolic compounds profile, carbohydrates and external fruit quality of the ‘Concorde’ pear (Pyrus communis L.) after bagging. Can. J. Plant Sci..

[50] Salta J., Martins A., Dos Santos R.G., Neng N., Nogueira J., Justino J., Rauter A.P. (2010). Phenolic composition and antioxidant activity of Rocha pear and other pear cultivars—A comparative study. J. Funct. Foods.

[51] Lin L.-Z., Harnly J.M. (2008). Phenolic compounds and chromatographic profiles of pear skins (Pyrus spp.). J. Agric. Food Chem..

[52] Ludwig I.A., Mena P., Calani L., Borges G., Pereira-Caro G., Bresciani L., Del Rio D., Lean M.E., Crozier A. (2015). New insights into the bioavailability of red raspberry anthocyanins and ellagitannins. Free Radic. Biol. Med..

[53] Piovesana A., Noreña C.P.Z. (2019). Study of Acidified Aqueous Extraction of Phenolic Compounds from Hibiscus sabdariffa L. calyces. Open Food Sci. J..

[54] Zamora-Ros R., Knaze V., Rothwell J.A., Hémon B., Moskal A., Overvad K., Tjønneland A., Kyrø C., Fagherazzi G., Boutron-Ruault M.-C. (2016). Dietary polyphenol intake in Europe: The European Prospective Investigation into Cancer and Nutrition (EPIC) study. Eur. J. Nutr..

[55] Hossain M.B., Rai D.K., Brunton N.P., Martin-Diana A.B., Barry-Ryan C. (2010). Characterization of phenolic composition in lamiaceae spices by lc-esi-ms/ms. J. Agric. Food Chem..

[56] Chaowuttikul C., Palanuvej C., Ruangrungsi N. (2020). Quantification of chlorogenic acid, rosmarinic acid, and caffeic acid contents in selected Thai medicinal plants using RP-HPLC-DAD. Braz. J. Pharm. Sci..

[57] Sasot G., Martínez-Huélamo M., Vallverdú-Queralt A., Mercader-Martí M., Estruch R., Lamuela-Raventós R.M. (2017). Identification of phenolic metabolites in human urine after the intake of a functional food made from grape extract by a high resolution LTQ-Orbitrap-MS approach. Food Res. Int..

[58] Cuadrado-Silva C.T., Pozo-Bayon M.A., Osorio C. (2016). Targeted Metabolomic Analysis of Polyphenols with Antioxidant Activity in Sour Guava (Psidium friedrichsthalianum Nied.) Fruit. Molecules.

[59] Trautvetter S., Koelling-Speer I., Speer K. (2009). Confirmation of phenolic acids and flavonoids in honeys by UPLC-MS. Apidologie.

[60] Petkovska A., Gjamovski V., Stanoeva J.P., Stefova M. (2017). Characterization of the Polyphenolic Profiles of Peel, Flesh and Leaves of Malus domestica Cultivars Using UHPLC-DAD-HESI-MSn. Nat. Prod. Commun..

[61] Chen G., Li X., Saleri F.D., Guo M. (2016). Analysis of Flavonoids in Rhamnus davurica and Its Antiproliferative Activities. Molecules.

[62] Raja M., Hernández-Revelles J., Hernández-Cassou S., Saurina J. (2014). Determination of polyphenols in the pear pulp matrix by solvent extraction and liquid chromatography with UV-Vis detection. Anal. Methods.

[63] Chung S.W. (2019). Anthocyanin Biosynthesis Associated with Skin Coloration in Highbush Blueberry Fruit During Ripening. Ph.D. Thesis.

[64] Tsao R., Yang R., Young A.J.C., Zhu H. (2003). Polyphenolic Profiles in Eight Apple Cultivars Using High-Performance Liquid Chromatography (HPLC). J. Agric. Food Chem..

[65] Dairpoosh F. (2011). Profile of Polyphenols in a European Diet. Ph.D. Thesis.

[66] Alvarez Arraibi A. (2018). Cosmeceutical Potential of Apple Pomace Phenolic Compounds: Development of a Natural-Based Dermal Hydrogel as Proof of Concept. Ph.D. Thesis.

[67] Lv Q., Luo F., Zhao X., Liu Y., Hu G., Sun C., Li X., Chen K. (2015). Identification of Proanthocyanidins from Litchi (Litchi chinensis Sonn.) Pulp by LC-ESI-Q-TOF-MS and Their Antioxidant Activity. PLoS ONE.

[68] De Pascual-Teresa S., Santos-Buelga C., Rivas-Gonzalo J.C. (2000). Quantitative Analysis of Flavan-3-ols in Spanish Foodstuffs and Beverages. J. Agric. Food Chem..

[69] Yuzuak S., Ballington J., Xie D.Y. (2018). Hplc-qtof-ms/ms-based profiling of flavan-3-ols and dimeric proanthocyanidins in berries of two muscadine grape hybrids flh 13-11 and flh 17-66. Metabolites.

[70] Arts I.C.W., Van De Putte B., Hollman P.C.H. (2000). Catechin Contents of Foods Commonly Consumed in the Netherlands. 1. Fruits, Vegetables, Staple Foods, and Processed Foods. J. Agric. Food Chem..

[71] Long W., Ye Z., Ping W., Zhen X., Zhuang W.J., Chong L., Qiong L. (2015). Rapid separation and identification of multiple constituents in vine tea by uflc system coupled with qtof-ms/ms. J. Pharm. Sci. Innov..

[72] Riethmüller E., Tóth G., Alberti Á., Végh K., Burlini I., Könczöl Á., Balogh G.T., Kéry Á. (2015). First characterisation of flavonoid-and diarylheptanoid-type antioxidant phenolics in corylus maxima by hplc-dad-esi-ms. J. Pharm. Biomed. Anal..

[73] Guijarro-Díez M., Nozal L., Marina M.L., Crego A.L. (2015). Metabolomic fingerprinting of saffron by LC/MS: Novel authenticity markers. Anal. Bioanal. Chem..

[74] Lantzouraki D.Z., Sinanoglou V.J., Tsiaka T., Proestos C., Zoumpoulakis P. (2015). Total phenolic content, antioxidant capacity and phytochemical profiling of grape and pomegranate wines. RSC Adv..

[75] Barbosa W.L.R., Peres A., Gallori S., Vincieri F.F. (2006). Determination of myricetin derivatives in Chrysobalanus icaco L. (Chrysobalanaceae). Rev. Bras. Farm..

[76] Zeng X., Su W., Zheng Y., Liu H., Li P., Zhang W., Liang Y., Bai Y., Peng W., Yao H. (2018). UFLC-Q-TOF-MS/MS-Based Screening and Identification of Flavonoids and Derived Metabolites in Human Urine after Oral Administration of Exocarpium Citri Grandis Extract. Molecules.

[77] Ma C., Dunshea F.R., Suleria H.A.R. (2019). LC-ESI-QTOF/MS Characterization of Phenolic Compounds in Palm Fruits (Jelly and Fishtail Palm) and Their Potential Antioxidant Activities. Antioxidants.

[78] Nogata Y., Ohta H., Yoza K.-I., Berhow M., Hasegawa S. (1994). High-performance liquid chromatographic determination of naturally occurring flavonoids in Citrus with a photodiode-array detector. J. Chromatogr. A.

[79] Lin L.-Z., Mukhopadhyay S., Robbins R.J., Harnly J.M. (2007). Identification and quantification of flavonoids of Mexican oregano (Lippia graveolens) by LC-DAD-ESI/MS analysis. J. Food Compos. Anal..

[80] Wang Y., Vorsa N., Harrington P.D.B., Chen P. (2018). Nontargeted Metabolomic Study on Variation of Phenolics in Different Cranberry Cultivars Using UPLC-IM-HRMS. J. Agric. Food Chem..

[81] Vuković N.L., Vukić M.D., Đelić G.T., Kacaniova M.M., Cvijović M. (2018). The investigation of bioactive secondary metabolites of the methanol extract of eryngium amethystinum. Kragujev. J. Sci..

[82] Pacifico S., Piccolella S., Lettieri A., Nocera P., Bollino F., Catauro M. (2017). A metabolic profiling approach to an Italian sage leaf extract (SoA541) defines its antioxidant and anti-acetylcholinesterase properties. J. Funct. Foods.

[83] Li X., Zhang J.-Y., Gao W.-Y., Wang Y., Wang H.-Y., Cao J.-G., Huang L.-Q. (2012). Chemical Composition and Anti-inflammatory and Antioxidant Activities of Eight Pear Cultivars. J. Agric. Food Chem..

[84] Pistelli L., Bertoli A., Gelli F., Bedini L., Ruffoni B., Pistelli L. (2012). Production of Curcuminoids in Different in vitro Organs of Curcuma longa. Nat. Prod. Commun..

[85] Dehghan H., Rezaee P., Aliahmadi A. (2020). Bioassay screening of 12 Iranian plants and detection of antibacterial compounds from Heracleum persicum using a TLC bioautography method. J. Liq. Chromatogr. Relat. Technol..

[86] Welke J.E., Zanus M.C., Lazzarotto M., Zini C.A. (2014). Quantitative analysis of headspace volatile compounds using comprehensive two-dimensional gas chromatography and their contribution to the aroma of Chardonnay wine. Food Res. Int..

[87] Zhang X., Yu Y., Cen Y., Yang D., Qi Z.-C., Hou Z., Han S., Chen Q., Liu K. (2018). Bivariate Correlation Analysis of the Chemometric Profiles of Chinese Wild Salvia miltiorrhiza Based on UPLC-Qqq-MS and Antioxidant Activities. Molecules.

[88] Kiokias S., Proestos C., Oreopoulou V. (2020). Phenolic acids of plant origin—A review on their antioxidant activity in vitro (o/w emulsion systems) along with their in vivo health biochemical properties. Foods.

[89] Liaudanskas M., Zymonė K., Viškelis J., Klevinskas A., Janulis V. (2017). Determination of the Phenolic Composition and Antioxidant Activity of Pear Extracts. J. Chem..

[90] Truong X.T., Park S.-H., Lee Y.-G., Jeong H.Y., Moon J.-H., Jeon T.-I. (2017). Protocatechuic Acid from Pear Inhibits Melanogenesis in Melanoma Cells. Int. J. Mol. Sci..

[91] Tanrioven D., Eksi A. (2005). Phenolic compounds in pear juice from different cultivars. Food Chem..

[92] Li X., Gao W.-Y., Huang L.-J., Zhang J.-Y., Guo X.-H. (2011). Antioxidant and Antiinflammation Capacities of Some Pear Cultivars. J. Food Sci..

