# Supplementary material for: A Comparative Investigation on Phenolic Composition, Characterization and Antioxidant Potentials of Five Different Australian Grown Pear Varieties
Source: Antioxidants (Basel). 2021 Jan 20;10(2):151. doi: 10.3390/antiox10020151 (PMC7909527; doi:10.3390/antiox10020151)
Supplement: Supplementary file 1 [file antioxidants-10-00151-s001.pdf]

antioxidants

Supplementary data

A Comparative Investigation on Phenolic Composition, Characterization and Antioxidant Potentials of Five Different Australian Grown PEAR Varieties

Zening Wang[1], Colin J. Barrow[2], Frank R. Dunshea[1,3] and Hafiz A.R. Suleria[1,2*]

[1] School of Agriculture and Food, Faculty of Veterinary and Agricultural Sciences, The University of Melbourne, Parkville 3010, VIC, Australia; zeningw1@student.unimelb.edu.au (Z.W.); fdunshea@unimelb.edu.au (F.R.D)

[2] Centre for Chemistry and Biotechnology, School of Life and Environmental Sciences, Deakin University, Waurn Ponds, VIC 3217, Australia; colin.barrow@deakin.edu.au (C.J.B)

[3] Faculty of Biological Sciences, The University of Leeds, Leeds LS2 9JT, UK

* Correspondence: Correspondence: hafiz.suleria@unimelb.edu.au; Tel.: +61-470-439-670

(a)

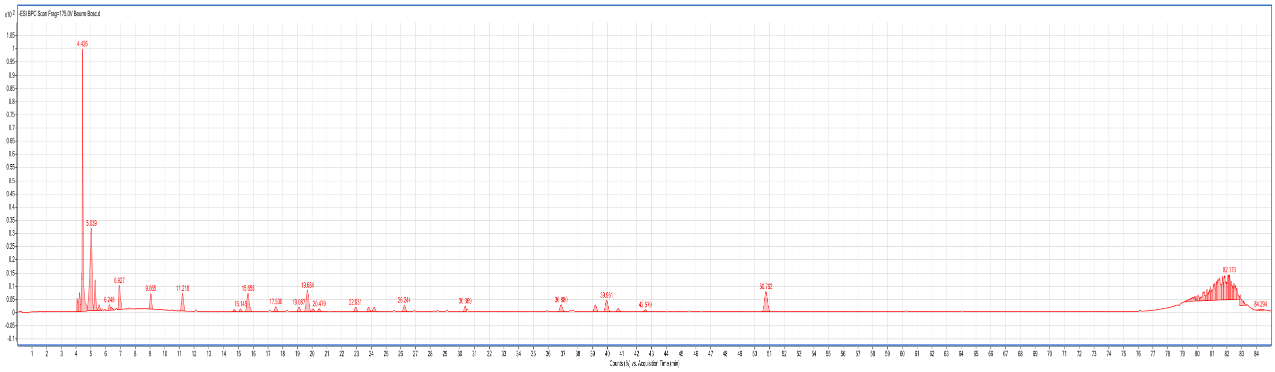

(b)

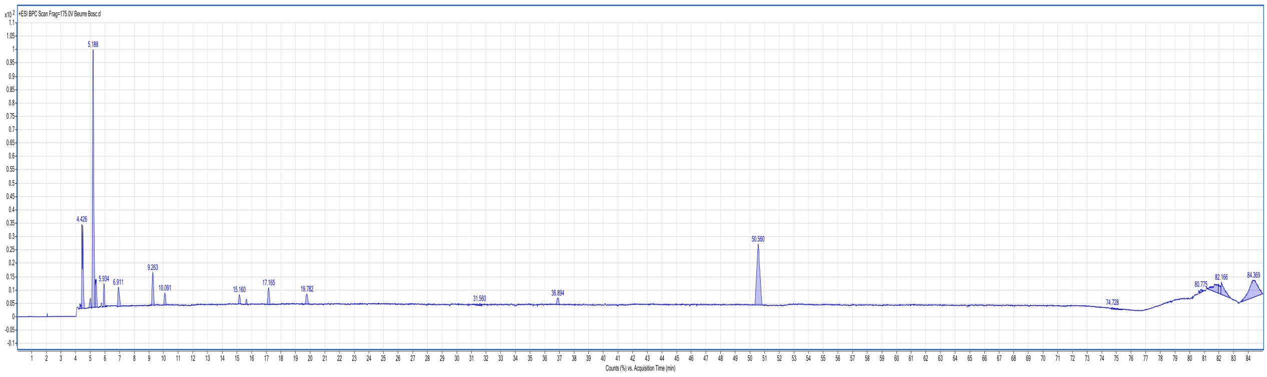

(c)

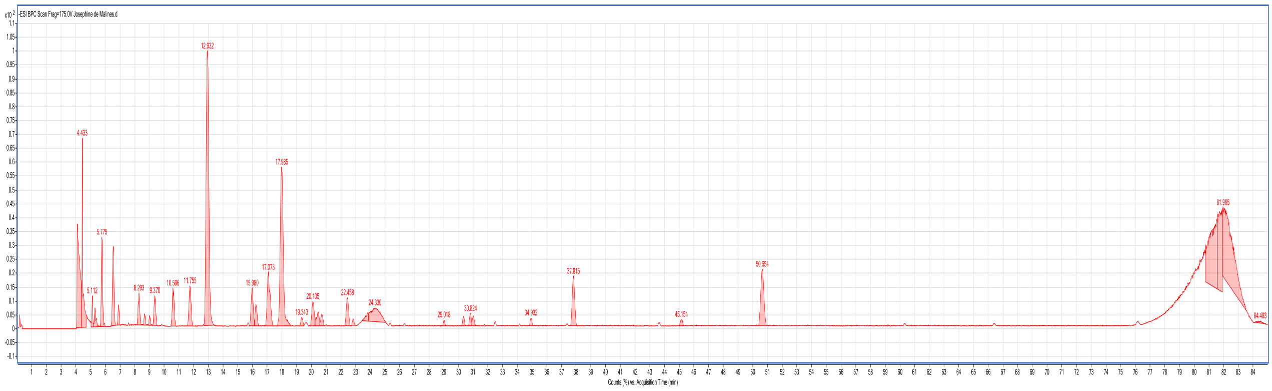
+ESI BPC Scan Frag=175.0V Josephine de Malines.d
Counts (%) vs. Acquisition Time (min)

(d)

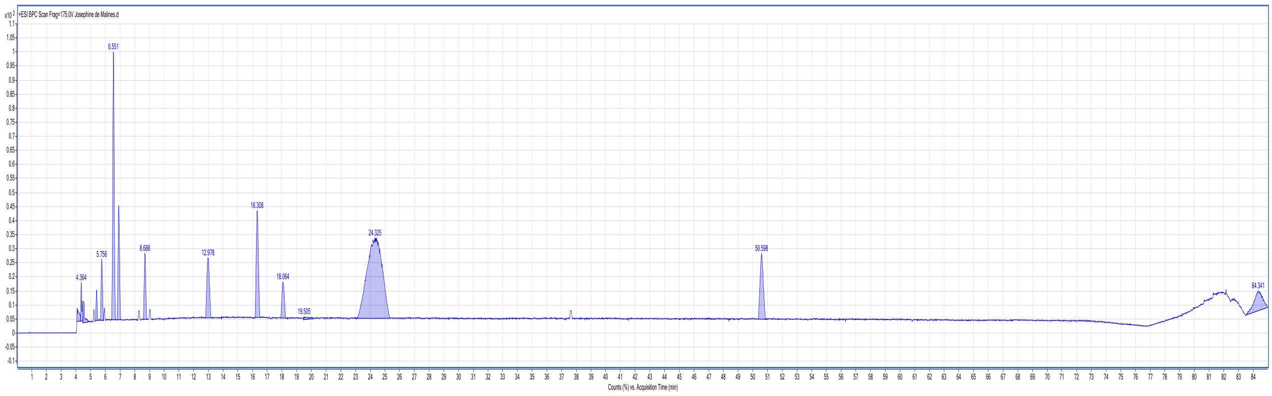
+ESI BPC Scan Frag=175.0V Josephine de Malines.d
Counts (%) vs. Acquisition Time (min)

(e)

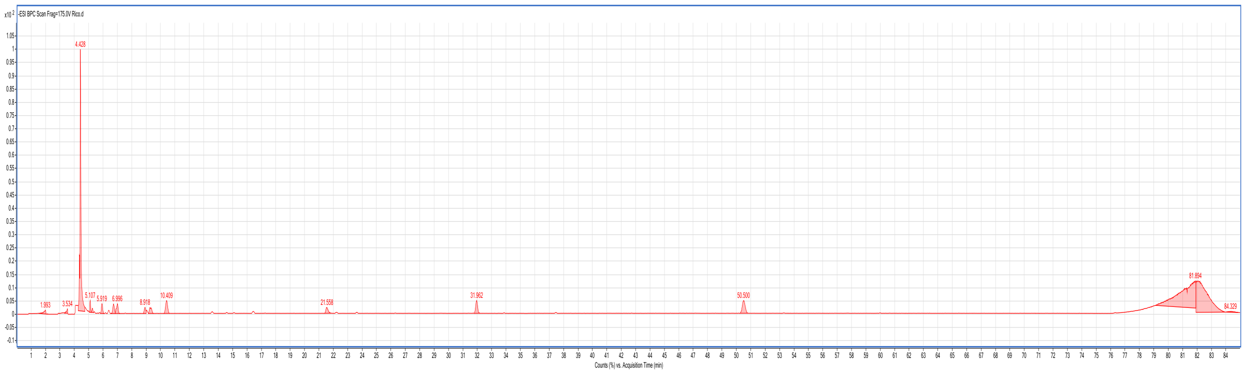
+ESI BPC Scan Frag=175.0V Ricco.d
Counts (%) vs. Acquisition Time (min)

(f)

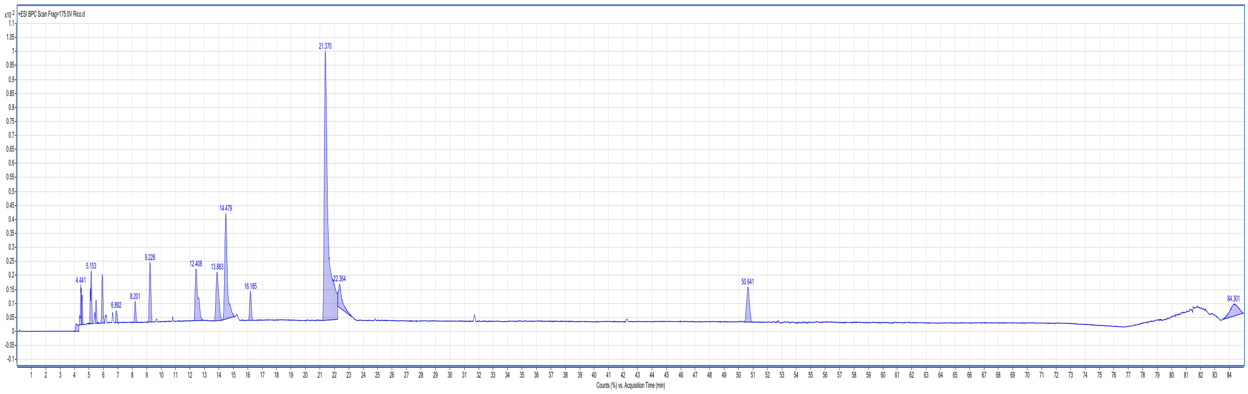
+ESI BPC Scan Frag=175.0V Ricco.d
Counts (%) vs. Acquisition Time (min)

(g)

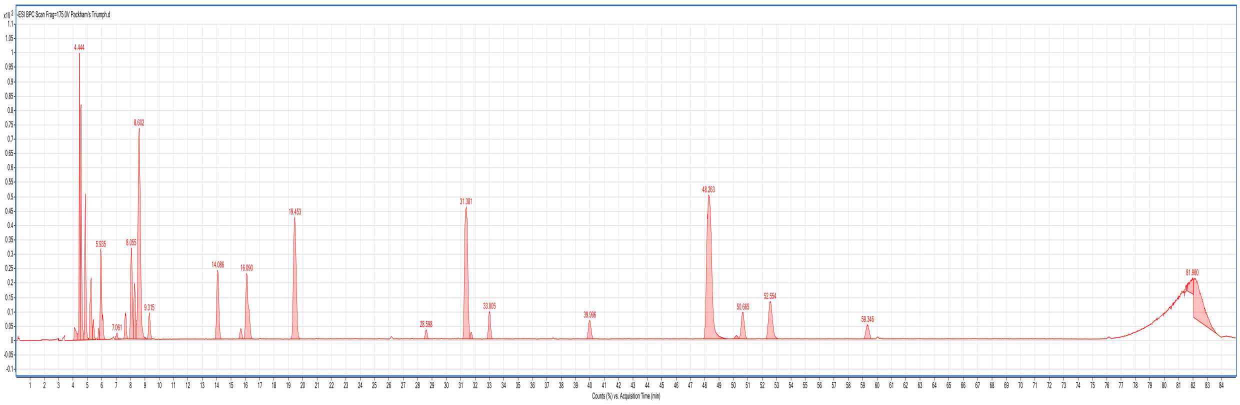
x10 2
-ESI BPC Scan Frag=175.0V Packham's Triumph.d
4.444
8.602
5.935
8.055
7.061
9.315
14.086
16.090
19.453
28.598
31.381
33.005
39.996
48.263
50.665
52.554
59.346
81.960
Counts (%) vs. Acquisition Time (min)

(h)

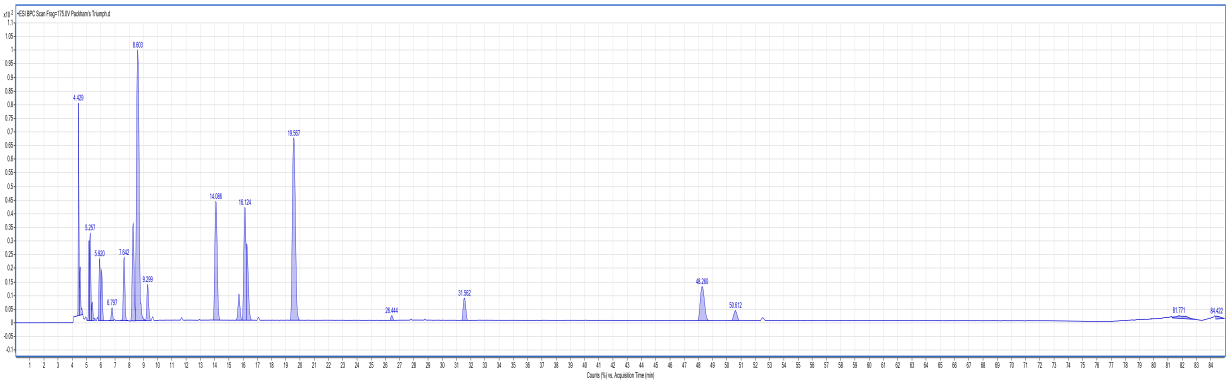
x10 2
+ESI BPC Scan Frag=175.0V Packham's Triumph.d
8.603
4.429
5.257
5.920
7.642
6.797
9.299
14.086
16.124
19.567
26.444
31.562
48.260
50.612
81.771
84.422
Counts (%) vs. Acquisition Time (min)

(i)

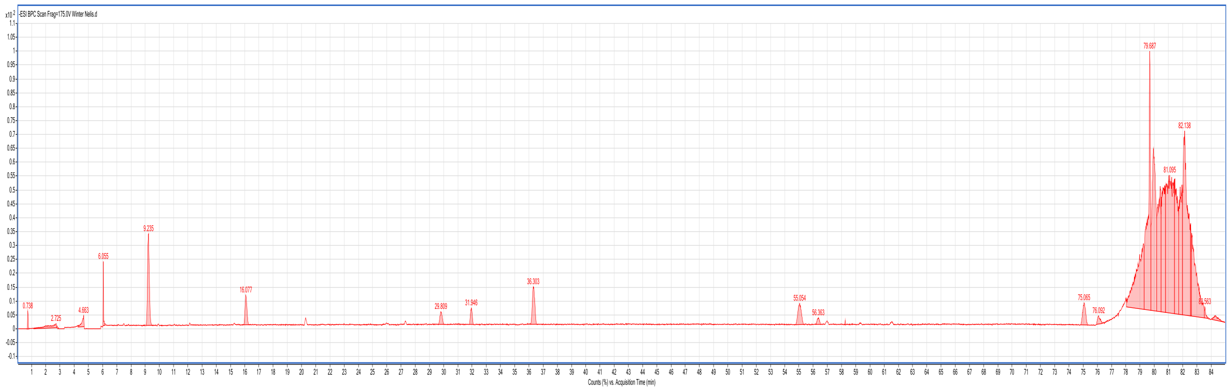

(j)

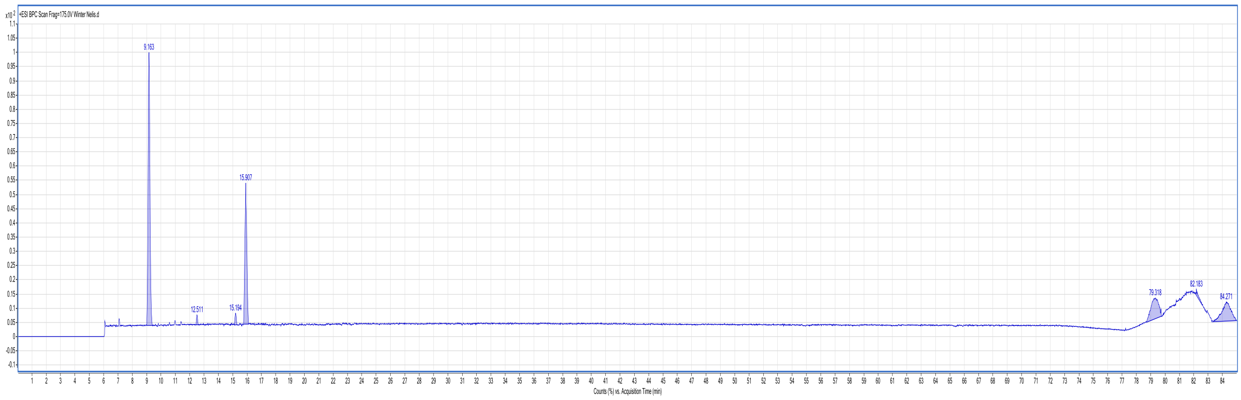

Figure S1. S): LC-ESI-QTOF-MS/MS basic peak chromatograph (BPC) for characterization of phenolic compounds of Australian grown pear varieties; **(a)** Beurre Bosc in negative ionization mode; **(b)** Beurre Bosc in positive ionization mode; **(c)** Josephine de Malines in negative ionization mode; **(d)** Josephine de Malines in positive ionization mode; **(e)** Rico in negative ionization mode; **(f)** Rico in positive ionization mode; **(g)** Packham's Triumph in negative ionization mode; **(h)** Packham's Triumph in positive ionization mode; **(i)** Winter Nelis in negative ionization mode; **(j)** Winter Nelis in positive ionization mode.

(a)

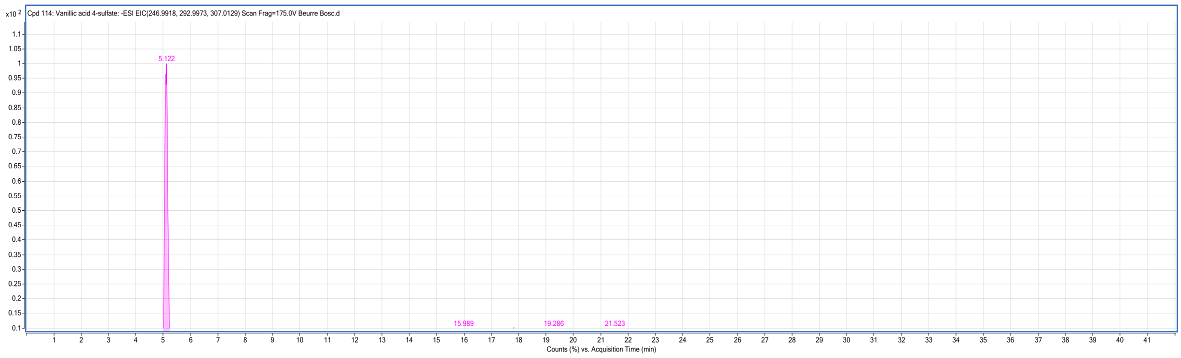

(b)

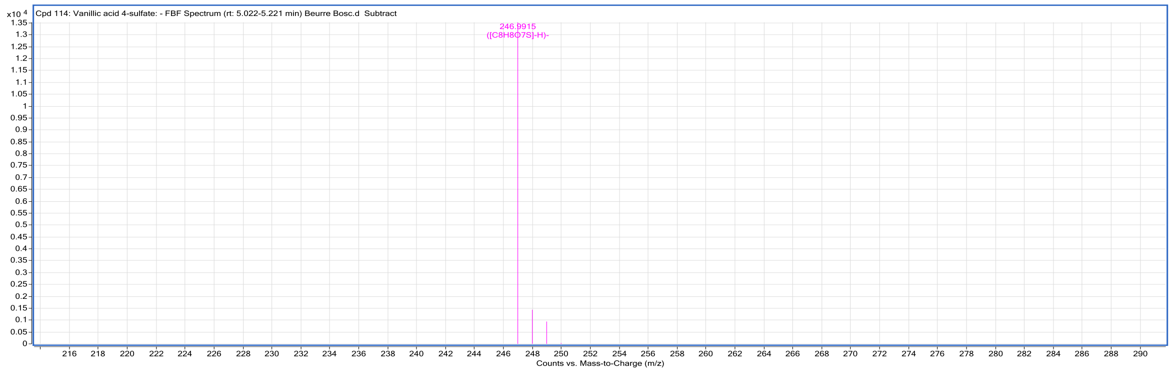
Cpd 114: Vanillic acid 4-sulfate: - FBF Spectrum (rt: 5.022-5.221 min) Beurre Bosc.d Subtract
246.9915
([C8H8O7S]-H)-
Counts vs. Mass-to-Charge (m/z)

(c)

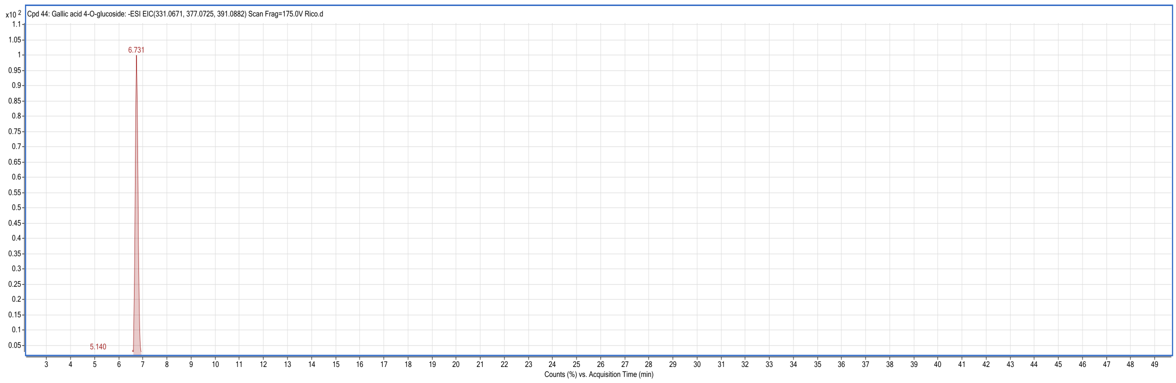
Cpd 44: Gallic acid 4-O-glucoside: -ESI EIC(331.0671, 377.0725, 391.0882) Scan Frag=175.0V Rico.d
6.731
5.140
Counts (%) vs. Acquisition Time (min)

(d)

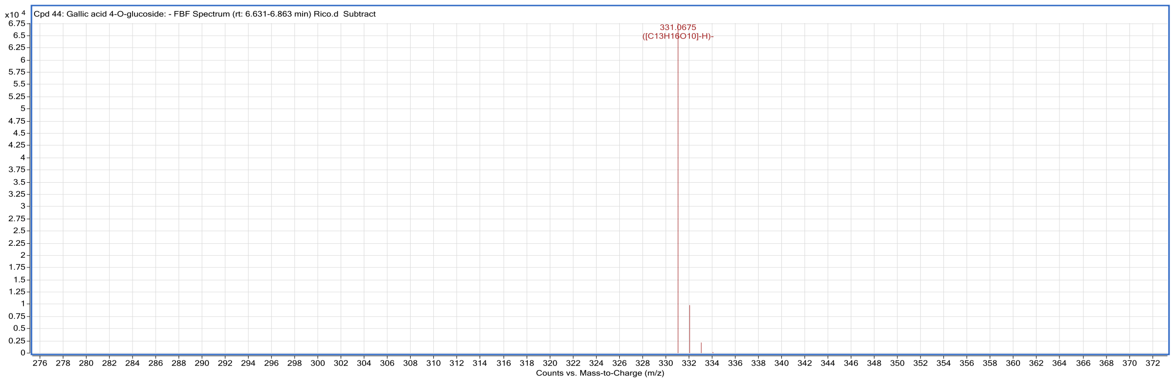
Cpd 44: Gallic acid 4-O-glucoside: - FBF Spectrum (rt: 6.631-6.863 min) Rico.d Subtract
331.0675
([C13H16O10]-H)-
Counts vs. Mass-to-Charge (m/z)

(e)

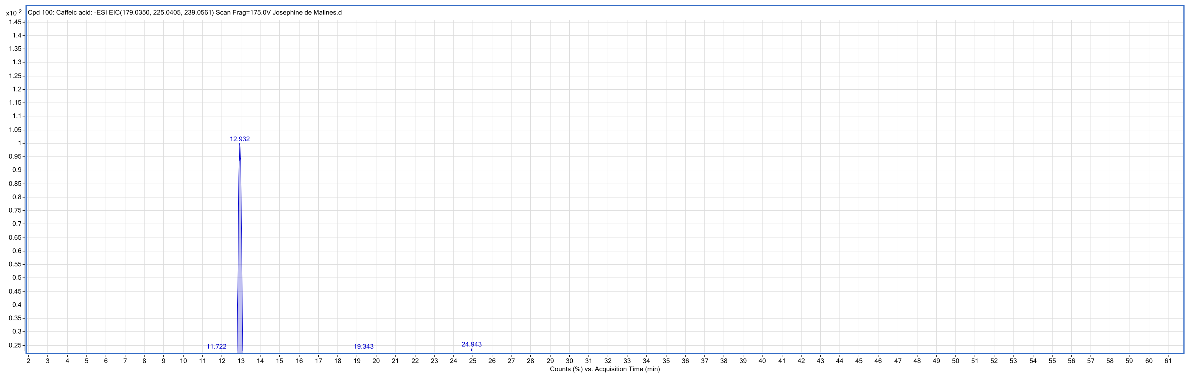
Cpd 100: Caffeic acid: -ESI EIC(179.0350, 225.0405, 239.0561) Scan Frag=175.0V Josephine de Malines.d
12.932
11.722
19.343
24.943
Counts (%) vs. Acquisition Time (min)

(f)

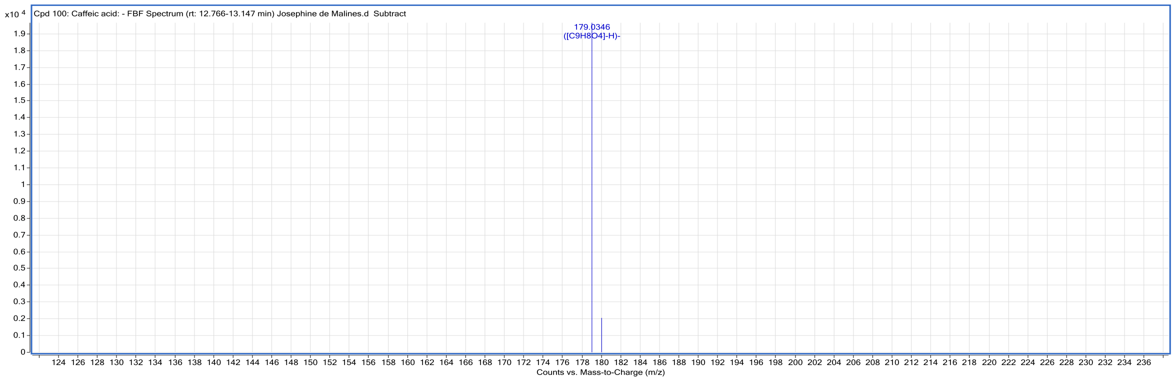
Cpd 100: Caffeic acid: - FBF Spectrum (rt: 12.766-13.147 min) Josephine de Malines.d Subtract
x10 4
179.0346
([C9H8O4]-H)-
Counts vs. Mass-to-Charge (m/z)

(g)

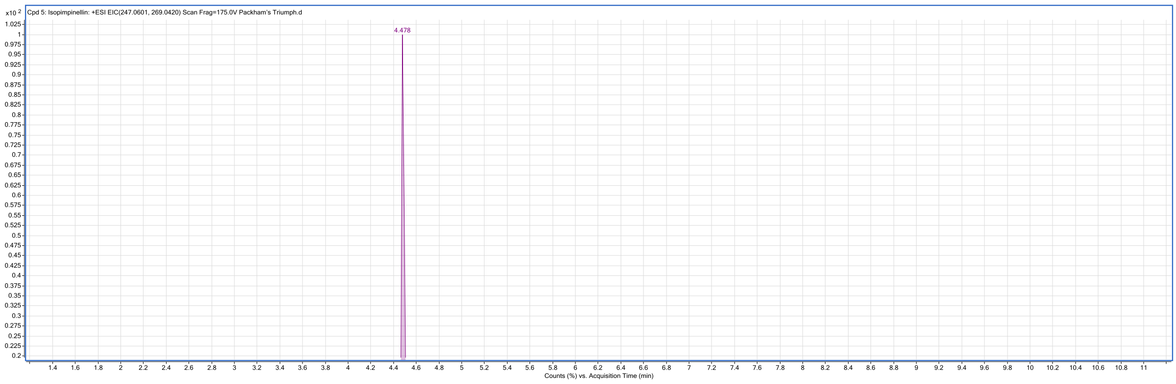
Cpd 5: Isopimpinellin: +ESI EIC(247.0601, 269.0420) Scan Frag=175.0V Packham's Triumph.d
x10 2
4.478
Counts (%) vs. Acquisition Time (min)

(h)

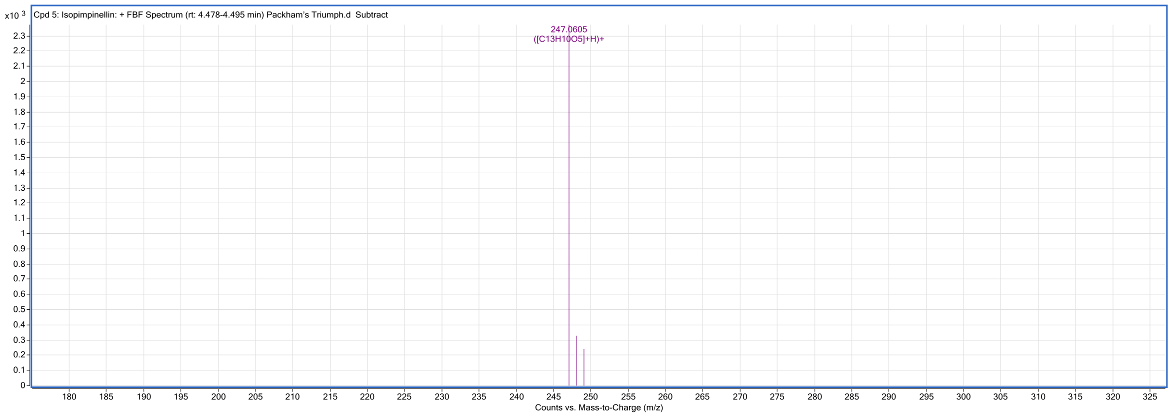
Cpd 5: Isopimpinellin: + FBF Spectrum (rt: 4.478-4.495 min) Packham's Triumph.d Subtract
x10 3
247.0605
([C13H10O5]+H)+
Counts vs. Mass-to-Charge (m/z)

(i)

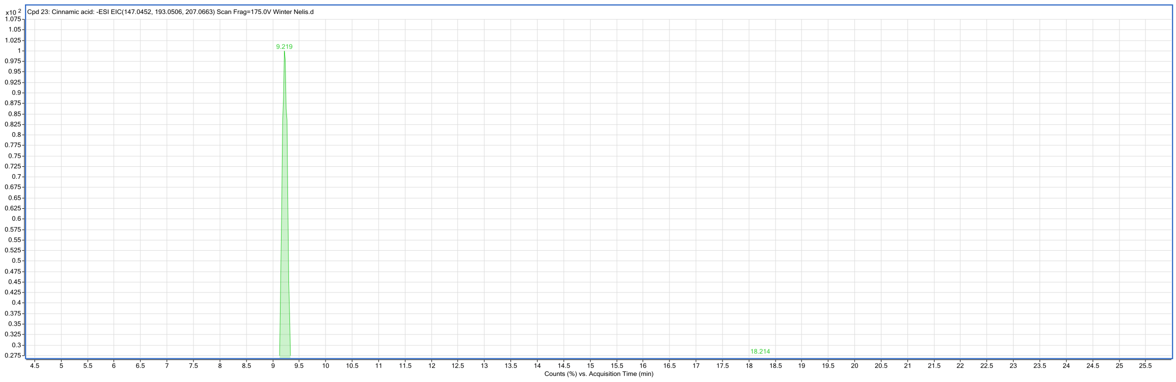
Cpd 23: Cinnamic acid: -ESI EIC(147.0452, 193.0506, 207.0663) Scan Frag=175.0V Winter Nelis.d
x10 2
9.219
18.214
Counts (%) vs. Acquisition Time (min)

(j)

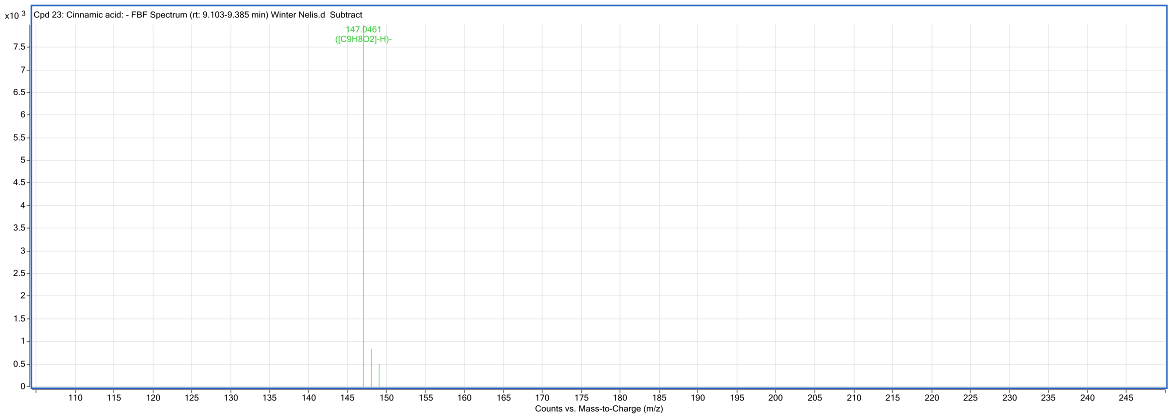

Figure S2. Extracted ion chromatogram and their mass spectrum. (a) A chromatograph of Vanillic acid 4-sulfate (Compound 1, Table 3), Retention time (RT = 5.122 min) in the negative mode of ionization (ESI$^-$/[M-H]$^-$) identified and characterized in Beurre Bosc pear sample; **(b)** Mass spectra of Vanillic acid 4-sulfate showing an observed *m/z* 246.9915 in Beurre Bosc; **(c)** A chromatograph of Gallic acid 4-*O*-glucoside (Compound 2, Table 3), Retention time (RT = 6.731 min) in the negative mode of ionization (ESI$^-$/[M-H]$^-$) identified and characterized in Rico pear sample; **(d)** Mass spectra of Gallic acid 4-*O*-glucoside showing an observed *m/z* 331.0675 in Rico; **(e)** A chromatograph of Caffeic acid (Compound 12, Table 3), Retention time (RT = 12.932 min) in the negative mode of ionization (ESI$^-$/[M-H]$^-$) identified and characterized in Josephine de Malines pear sample; **(f)** Mass spectra of Caffeic acid showing an observed *m/z* 179.0346 in Josephine de Malines; **g)** A chromatograph of Isopimpinellin (Compound 62, Table 3), Retention time (RT = 4.478 min) in the positive mode of ionization (ESI$^+$/[M+H]$^+$) identified and characterized in Packham's Triumph pear sample; **(h)** Mass spectra of Isopimpinellin showing an observed *m/z* 247.0605 in Packham's Triumph; **(i)** A chromatograph of Cinnamic acid (Compound 10, Table 3), Retention time (RT = 9.219 min) in the negative mode of ionization (ESI$^-$/[M-H]$^-$) identified and characterized in Winter Nelis pear sample; **(j)** Mass spectra of Cinnamic acid showing an observed *m/z* 147.0461 in Winter Nelis.
